# Developing Familiarity in a New Duo: Rehearsal Talk and Performance Cues

**DOI:** 10.3389/fpsyg.2021.590987

**Published:** 2021-03-16

**Authors:** Jane Ginsborg, Dawn Bennett

**Affiliations:** ^1^Centre for Music Performance Research, Royal Northern College of Music, Manchester, United Kingdom; ^2^Assistant Provost, Bond University, Robina, QLD, Australia

**Keywords:** cognitive processes, memorization, preparation for performance, socio-emotional processes, verbal communication

## Abstract

**Context and Aims:** Social and cognitive processes underlying individual classical musicians' and duo performers' preparation for performance have been explored using longitudinal case studies. Social processes can be inferred from *rehearsal talk* and recent studies have focused on its content and nature. Cognitive processes can be inferred from score annotations representing musicians' thoughts while practicing, rehearsing (*rehearsal features*), and playing or singing from memory (*performance cues*). We report three studies conducted by two practitioner-researchers: (1) of rehearsal talk; (2) of rehearsal features and thoughts while performing; and (3) a triangulation (as it were) of the two kinds of data to gauge the potential for rehearsal talk to predict the use of performance cues.

**Methods:** A singer and viola player formed a new duo to prepare two songs, new to them both, for two performances on the same day and a third performance 10 months later. Their practice and rehearsal sessions, over the course of seven days, were recorded and transcribed. The musicians annotated copies of the scores after rehearsing and after each performance. Each musician performed one of the two songs from memory. First, verbal data were coded and analyzed using two frameworks for categorizing socio-emotional interactions and musical dimensions, respectively. Second, their annotations were categorized and compared, and finally the frameworks were combined so that correlations between rehearsal talk and performance cues could be calculated.

**Results:** The musicians' verbal interactions were positive and task-related; significant changes over time were observed only in the extent to which they showed solidarity toward each other. Analysis of their annotations illustrates similarities and differences between their attention to specific features of the music while rehearsing and performing, particularly from memory. Rehearsal talk predicted performance cues in the third performance, but not the first or second.

**Conclusion:** Musicians' talk cannot be assumed to reflect musicians' actions. The study of musicians' verbal interactions may be less useful for determining cognitive than social processes underlying preparation for performance. Nevertheless, the study provides a detailed snapshot of classical musicians' “real world” preparation for performance, highlighting the role of spontaneity in performance, and underlining differences between what happens in the studio and what can happen on stage.

## Introduction

When two musicians come together for the first time to rehearse a piece of music that is new to them both, they have to develop two kinds of familiarity: first with each other, as duo partners (*social familiarity*) and, second, with the music. Typically they will have practiced the music independently before they rehearse together, but their conceptions of the music may be very different, although—according to the conventions of Western classical music—when they come to perform it in public they must present what appears, at least, to be a unanimous interpretation of their joint understanding of the composer's intentions.^1^

There is a great deal of literature on the cognitive and social processes that underlie ensemble rehearsal and performance (see Keller et al., 2016; Ginsborg, 2017; McCaleb, 2017; MacRitchie et al., 2018, for reviews). Topics include empathetic creativity in the music making of jazz sextets and string quartets (Seddon, 2012), non-verbal communication between the members of piano duos (e.g., Williamon and Davidson, 2002; Blank and Davidson, 2007; Bishop and Goebl, 2020) and string quartets (e.g., Hospelhorn and Radinsky, 2017), interpersonal coordination (e.g., Novembre and Keller, 2018), for example between members of a small vocal group (D'Amario et al., 2020), and social familiarity (e.g., King, 2013).

Social familiarity has previously been manipulated in an experiment designed to explore verbal (Ginsborg and King, 2012) and non-verbal communication (King and Ginsborg, 2011) between members of two established student singer-pianist duos and two established professional singer-pianist duos. In each of three sessions the musicians rehearsed and performed a song previously unknown to them, first with their regular duo partner, second with a new same-expertise partner, and third (in two cases) with a new different-expertise partner. The established duos used more, and a wider range of bodily gestures when rehearsing with their regular partners than with new partners, and the behaviors of the newly formed duos became more synchronous over the course of their rehearsals. The professional duos also talked less and sang and played more.

Singer-pianist duos differ from many other kinds of duo in that the singer is often expected to perform from memory. Again, there is a great deal of literature on the cognitive processes underlying musicians' memorization strategies and recall from memory (see Chaffin et al., 2016; Ginsborg, in press, for reviews). A growing body of research on the development of *content-addressable memory* (the result of deliberate, thoughtful practice), as opposed to *serial cuing* (formerly referred to as “associative chaining,” the result of repetition), has drawn on the findings of longitudinal case studies with practitioner-researchers: musicians who have tracked their learning and memorization over days, weeks, and years; these include the pianists Gabriela Imreh (e.g., Chaffin et al., 2002) and Cristina Capparelli Gerling (Chaffin et al., 2013), the cellist Tânia Lisboa (e.g., Lisboa et al., 2018) and the singer Jane Ginsborg (e.g., Ginsborg and Chaffin, 2011; Ginsborg et al., 2012). These findings suggest that during the course of practice and rehearsal, musicians form mental representations or maps of the works they are preparing to perform from memory, with landmarks for retrieval. Each map can be thought of as a *hierarchical retrieval organization* and each landmark as a potential *performance cue*. According to performance cue theory as originally formulated by Chaffin et al. (2002), performance cues are a subset of *practice* or *rehearsal features*: the features of the music to which the musician attends while practicing independently and rehearsing with a partner. Ginsborg et al. (2012) presented preliminary evidence suggesting that spontaneous thoughts in performance could also function as performance cues in subsequent performances, but this warranted further study.

The present investigation was designed to bring together these two lines of enquiry:

1) the role and development of social familiarity between the members of a newly formed duo and the development of familiarity with the work to be performed; and
2) the role and development of rehearsal features in the course of preparation for performance, and spontaneous thoughts while performing, as potential performance cues.

Accordingly, this article reports a longitudinal case study of a singer and a viola player, the first and second author, respectively, who prepared to perform two songs that were unfamiliar to both of them, after seven days during which they practiced independently and rehearsed together daily. All practice sessions and rehearsals were audio-recorded and both verbal and musical utterances were transcribed verbatim. The musicians gave three performances of the work: two on the same day, at the end of the week, and a third performance 10 months later, after a single short rehearsal. The viola player memorized and performed the first song from memory while the singer read from the score; the singer memorized and performed the second song from memory while the viola player read from the score. They both annotated copies of the score, independently and together, at the end of the initial rehearsal period and after each performance to indicate their rehearsal features and potential performance cues.

A multi-strategy approach was taken, involving three analyses. The first (Study 1) was a content analysis of rehearsal talk. A framework adapted from Ginsborg and King (2012) was used to identify the musical dimensions and rehearsal strategies to which they referred and Interaction Process Analysis (Bales, 1999) was used to explore socio-emotional interactions between the musicians. Study 2 investigated the extent to which rehearsal features and spontaneous thoughts while performing are retained as performance cues over time. Study 3 represents a triangulation of the results of Studies 1 and 2 by exploring potential relationships between rehearsal talk, in relation to musical dimensions, and performance cues.^2^

### Material and Methods (Studies 1–3)

The two authors are both highly experienced musicians who have performed with their own duo partners (pianists) for many years. Despite being familiar with each other's practice-based and non-practice-based research (in the fields of music psychology and music education, broadly defined), they had not rehearsed or performed together before commencing the present study. The first author is a singer, the second a viola player.

The criteria for selecting the music were that it should be for voice and viola duo (a comparatively rare combination), unknown to both musicians, and of a suitable length to be rehearsed over the course of a week and performed at the end of the rehearsal period. *From Kipling* by Boris Tchaikovsky (1925–1996) (Tchaikovsky, 1994) consists of two songs, settings of texts by Rudyard Kipling, “Far-off Amazon” (“Amazon”) and “Homer,” loosely translated into Russian (see Appendix A for text, translation, and original poems). The duration of “Amazon” is 2 min 10 s and that of “Homer” 2 min 58 s. The viola player memorized the first song and played it from memory in performance, while the singer memorized the second song and sang it from memory.

As shown in Table 1, the two musicians practiced independently and/or rehearsed together (twice, on two days) on seven consecutive days, giving two performances of the work to a small invited audience on the last day. After the final rehearsal the musicians annotated copies of their scores to indicate the locations of their rehearsal features, and after each performance they annotated copies of their scores to indicate the locations of their performance cues; 10 months later they held another short rehearsal and gave a third performance of the work, in the context of a concert of music for singer, viola and piano, using the same procedure to record thoughts during performance. All practice sessions, rehearsals and performances were audio-recorded using the musicians' smartphones; data were stored as .wav files and subsequently transcribed for analysis.

**Table 1:** Practice and rehearsal time (both songs).

**Date (2014)**	**Individual practice time**	**Rehearsal time**
24 March	1 h 17 m	20 m
25 March	0 h 17 m	34 m
26 March	0 h 33 m	
27 March	0 h 57 m	26 m
		37 m
28 March	1 h 04 m	
29 March		27 m
30 March		33 m
		27 m
Total	4 h 08 m	3 h 24 m
**Rehearsal feature reports and performances**		
30 March	Performance 1 + Thoughts 1	
30 March	Performance 2 + Thoughts 2	
10 January 2015	Short rehearsal and performance + Thoughts 3	

## Study 1

### Introduction

Verbal communication between a singer and a pianist was explored by Ginsborg et al. (2006) and between the members of four singer-pianist duos by Ginsborg and King (2012) using content analyses of rehearsal talk. In both studies the content analyses were guided by a framework of *musical dimensions* incorporating references to *basic/structural, interpretive* (including *expressive*) rehearsal features initially developed by Chaffin et al. (2002: see Table 2) and *rehearsal strategies*. The verbal utterances of the singers and pianists in Ginsborg and King's study were also coded using the Interaction Process Analysis framework developed by Bales (1999: see Table 3).

**Table 2:** Musical dimensions framework.

**Dimensions**	**Features of the music and its performance**
Basic/structural	Pitch, tempo, technique, breath, ensemble, harmony, composition, notation, meter, entries, structure, switch (where the same music can lead in one of two or more directions)
Interpretive	Rubato, dynamics, words, tempo, phrasing, articulation, color, expressive (conveying interpretative intentions to the audience)
Rehearsal strategy	Whole song, repeat section etc., learning, slow or speed tempo, rehearse verse, rehearsal (general), prepare for performance, memory.

**Table 3:** Interaction process framework.

Socio-emotional	Positive	Agree
		Show solidarity
		Tension release
	Negative	Disagree
		Show antagonism
		Show tension
Task	Answers	Give opinion
		Provide orientation
		Make suggestion
	Questions	Ask for opinion
		Ask for orientation
		Ask for suggestion

#### Aims of the Content Analysis

The aims of the analysis reported as Study 1 were to replicate certain elements of Ginsborg and King's (2012) study by exploring the potential effects of expertise and familiarity on two musicians at similar levels of expertise as musicians: they were of similar ages, had similar experiences of secondary and tertiary music education and training, and had both enjoyed professional careers as musicians before undertaking doctoral research and establishing themselves as academics. The singer had greater expertise than the viola player, however, in performing *from memory*, as this is expected of singers particularly when giving song recitals, but not of string players within larger ensembles, which was the context in which the viola player had most experience. In terms of familiarity, the musicians had not worked together before when they started rehearsing, but familiarity between them developed over the week during which they rehearsed daily, from Rehearsal 1 to Rehearsal 7.

#### Research Questions

The overarching research question asked what can be learned about the cognitive and social processes underlying preparation for performance from the observation of what musicians talk about, and how they talk about it, when they rehearse. The following specific questions were addressed:

1. What proportion of the rehearsals of the two songs consisted of talk rather than playing and/or singing?
2. In each rehearsal of each song, how many conversations between episodes of singing and playing (*verbal exchanges*) took place, consisting of how many utterances, and who initiated each exchange?
3. What was the *content* and *nature* of the musicians' rehearsal talk?
4. To what extent could any differences between the musicians' rehearsal talk be attributed to (a) instrument (voice vs. viola), (b) whether or not they were memorizing the song being rehearsed, (c) the song itself, and/or (d) time, that is, over the course of rehearsals?

### Results

#### Proportion of the Rehearsals of the Two Songs That Consisted of Talk Rather Than Playing and/or Singing

As shown in Table 4, the two songs were rehearsed in seven rehearsal sessions ranging between 19 and 37 min in length and lasting a total of 3 h and 24 min, of which almost exactly half (1 h 42 min) consisted of talking. “Amazon” was rehearsed in all sessions (1 h 51 min, of which 1 h 02 min consisted of talking) and “Homer” in all but one session (1 h 32 min of which 39 min consisted of talking). Thus, “Amazon” received 8% more rehearsal time than “Homer” (111/204*100 = 54% vs. 93/204*100 = 46%) of which a larger proportion (62/111*100 = 56% vs. 40/93*100 = 43%) consisted of talking.

**Table 4:** Length of talk in rehearsals of each song.

	**Amazon (2 m 10 s)**		**Homer (2 m 58 s)**		**Total**	
**Session (date)**	**Overall length**	**Talk**	**Overall length**	**Talk**	**Overall length**	**Talk**
1 (24 March)	14 m 40 s	5 m 46 s				
			4 m 43 s	0 m 47 s	19 m 23 s	6 m 33 s
2 (25 March)	18 m 38 s	11 m 56 s				
			15 m 0 s	8 m 26 s	33 m 38 s	20 m 22 s
3 (26 March)	26 m 20 s	17 m 40 s			26 m 20 s	17 m 40 s
4 (27 March)	9 m 13 s	5 m 15 s				
			27 m 52 s	11 m 34 s	37 m 05 s	16 m 49 s
5 (28 March)			16 m 30 s	6 m 30 s		
	10 m 33 s	5 m 14 s			27 m 03 s	11 m 44 s
6 (29 March)	24 m 06 s	13 m 11 s				
			8 m 48 s	3 m 18 s	32 m 54 s	16 m 29 s
7 (30 March)			13 m 21 s	6 m 03 s		
	7 m 20 s	2m 55s				
			6 m 27 s	3 m 0 s		
			19 m 48 s	9 m 03 s	27 m 08 s	11 m 58 s
Total	110 m 50 s	61 m 57 s	92 m 41 s	39 m 38 s	203 m 31 s	101 m 35 s

#### Verbal Exchanges, Utterances, and Initiators

Numbers of verbal exchanges, utterances, minimum and maximum numbers (range) of utterances in each exchange, mean number of utterances per exchange, and the numbers and percentages of exchanges initiated by each musician in each session are presented in Table 5. “Amazon” received not only more rehearsal time but more rehearsal talk, with a mean of 10 exchanges per session and 14 utterances per exchange, while there was a mean of 8 exchanges per session for “Homer” with 10.75 utterances per exchange. The singer initiated 62.7% of all exchanges, 65.7% in “Amazon” rehearsals and 58.3% in “Homer” rehearsals.

**Table 5:** Verbal exchanges, utterances, and initiators.

**Session**	**Exchanges**	**Number of utterances**	**Range**	**Mean utterances/exchange**	**Singer initiate**	**Viola player initiate**
**Amazon (viola from memory)**						
1	18	113	1–17	6.3	15 (83.3%)	3 (16.7%)
2	16	190	1–39	11.9	12 (81.3%)	3 (19.7%)
3	12	269	2–83	22.4	7 (58.3%)	5 (41.7%)
4	8	101	3–25	12.6	4 (50%)	4 (50%)
5	6	49	3–16	8.2	2 (33.3%)	4 (66.7%)
6	8	206	9–47	25.8	4 (50%)	4 (50%)
7	2	50	10–40	25.0	1 (50%)	1 (50%)
All	70	978	1–83	14.0	46 (65.7%)	24 (34.3%)
**Homer (singer from memory)**						
1	4	24	1–14	6	1 (25%)	3 (75%)
2	7	88	1–44	12.6	4 (57.1%)	3 (42.9%)
4	14	189	3–21	13.5	9 (64.3%)	5 (35.7%)
5	9	92	1–25	10.2	4 (44.4%)	5 (55.6%)
6	5	52	3–23	10.4	4 (80%)	1 (20%)
7	9	71	1–23	7.89	6 (66.7%)	3 (33.3%)
All	48	516	1–44	10.75	28 (58.3%)	20 (41.7%)
**Both songs**						
1	22	137	1–17	6.2	16 (72.7%)	6 (27.3%)
2	23	278	1–44	12.1	17 (73.9%)	6 (26.1%)
3	12	269	2–83	22.4	7 (58.3%)	5 (41.7%)
4	22	290	3–25	13.2	13 (59.1%)	9 (40.9%)
5	15	141	1–25	9.4	6 (40%)	9 (60%)
6	13	258	3–47	19.8	8 (61.5%)	5 (38.5%)
7	11	121	1–40	11.0	7 (63.6%)	4 (36.3%)
All	118	1,494	1–83	13.45	74 (62.7%)	(37.3%)

#### Content and Nature of Rehearsal Talk

Before rehearsal talk in all seven rehearsals was analyzed, the two authors independently coded transcripts of two rehearsals, comprising 20.2% of the complete dataset. The two sets of codes were checked for inter-observer reliability using Cohen's *kappa*. A total of 207 Interaction Process codes was assigned to 302 utterances with a reliability of 0.93 and 206 musical dimensions codes were assigned to the same utterances with a reliability of 0.98. The first author then coded the remainder of the transcripts, the coding was checked by the second author, and disagreements were resolved following discussion.

Table 6 presents the numbers of utterances made by each musician and both musicians during rehearsals of each song that were coded using the two frameworks.

**Table 6:** Utterances coded using the interaction process and musical dimensions frameworks.

	**Singer**	**Viola player**	**Both**
**Interaction process**			
Amazon	352	262	614
Homer	165	163	334
**Musical dimensions**			
Amazon	252	84	336
Homer	182	64	246

##### Musical Dimensions and Rehearsal Strategies

**Figure 1:**
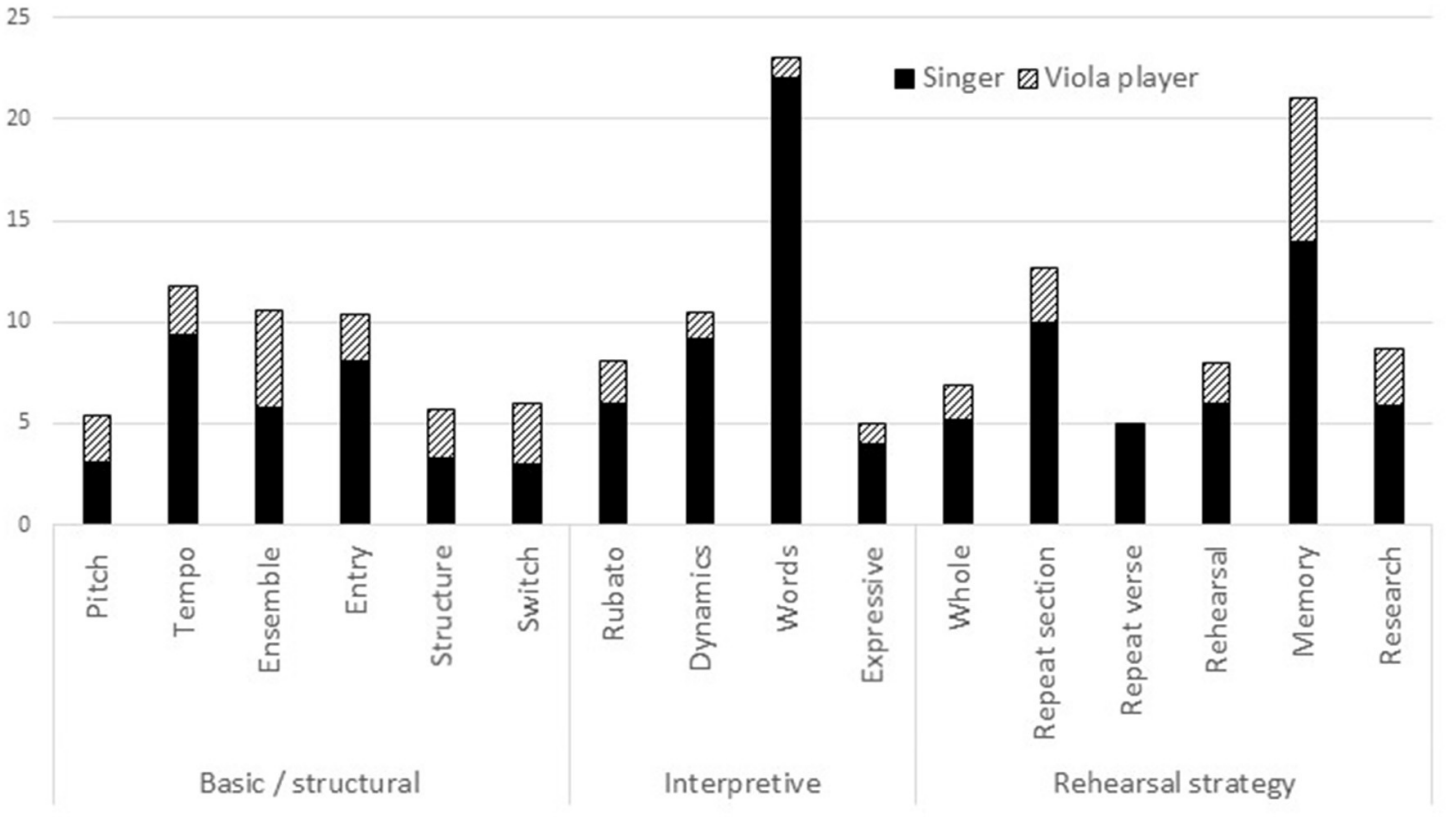
Content of rehearsal talk (percentages of utterances).

The numbers of utterances made in each sub-category of the *basic* and *interpretive dimensions* and *rehearsal strategies* by each musician in all rehearsals of both songs were calculated as percentages of utterances in all rehearsals to which musical dimensions codes had been assigned. For example, the two musicians' 336 utterances during seven rehearsals of “Amazon” included 22 references to *tempo* (6.55%) made by the singer and four by the viola player (1.19%). Their 246 utterances during six rehearsals of “Homer” included seven references to *tempo* by the singer (2.85%) and three by the viola player (1.19%). Thus, as shown in Figure 1, 11.8% of all utterances referred to *tempo*. Other basic features mentioned comparatively often were *ensemble* (10.6%) and *entries* (10%). The most frequently mentioned interpretive features were *dynamics* (10%) and *words* (23%), while the most frequently mentioned rehearsal strategies were *repeat section* (13%) and (work on) *memory* (21%).

##### Interaction Process Analysis

**Figure 2:**
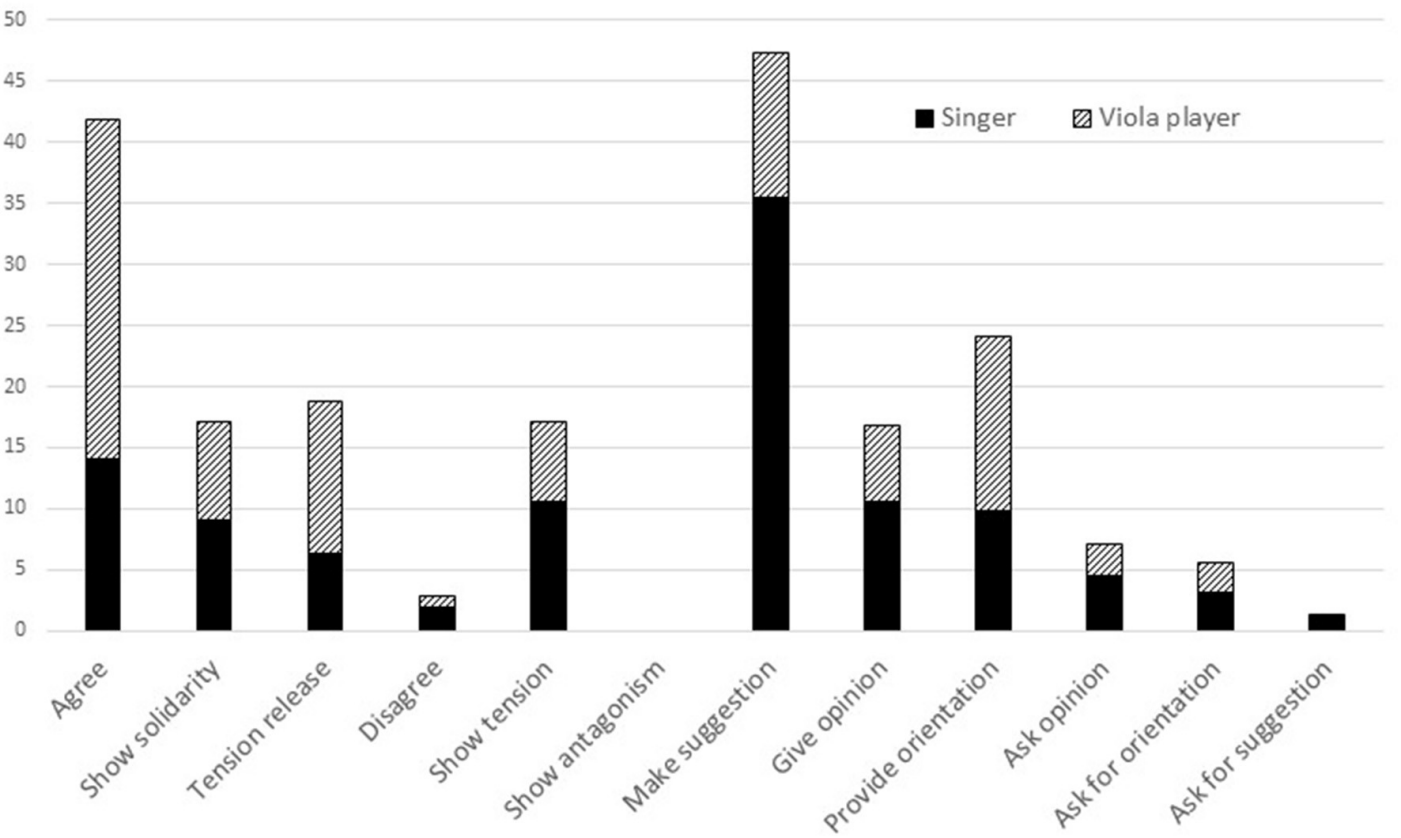
Nature of rehearsal talk (percentages of utterances).

The numbers of utterances made in each sub-category of the *positive* and *negative socio-emotional* categories and the *answer* and *question task* categories (see Table 3) by each musician in all rehearsals were calculated as percentages of all utterances in all rehearsals to which Interaction Process codes had been assigned. As shown in Figure 2, the musicians were most likely to *make suggestion*s (47.4%), *agree* (41.8%) and *provide orientation* (24.2%); *disagreements* were rare (2.8%) and there was no *antagonism* between them.

#### Differences Between the Musicians' Rehearsal Talk

Comparisons were made between the content of the musicians' utterances in the seven categories referring to musical dimensions in more than 10% of utterances per song (for pragmatic reasons): those coded as basic (*tempo, ensemble*, and *entry*); those coded as interpretive (*dynamics* and *words*); the rehearsal strategies *repeat section* and (work on) *memory*. Comparisons were also made between the nature of musicians' utterances in the seven categories of interaction process in more than 15% of utterances per song (again, for pragmatic reasons): each of the three positive socio-emotional categories *agree, show solidarity*, and *tension release*; each of the three task answer categories *make suggestion, give opinion*, and *provide orientation*; and one negative socio-emotional category *show tension*.

**Figure 3:**
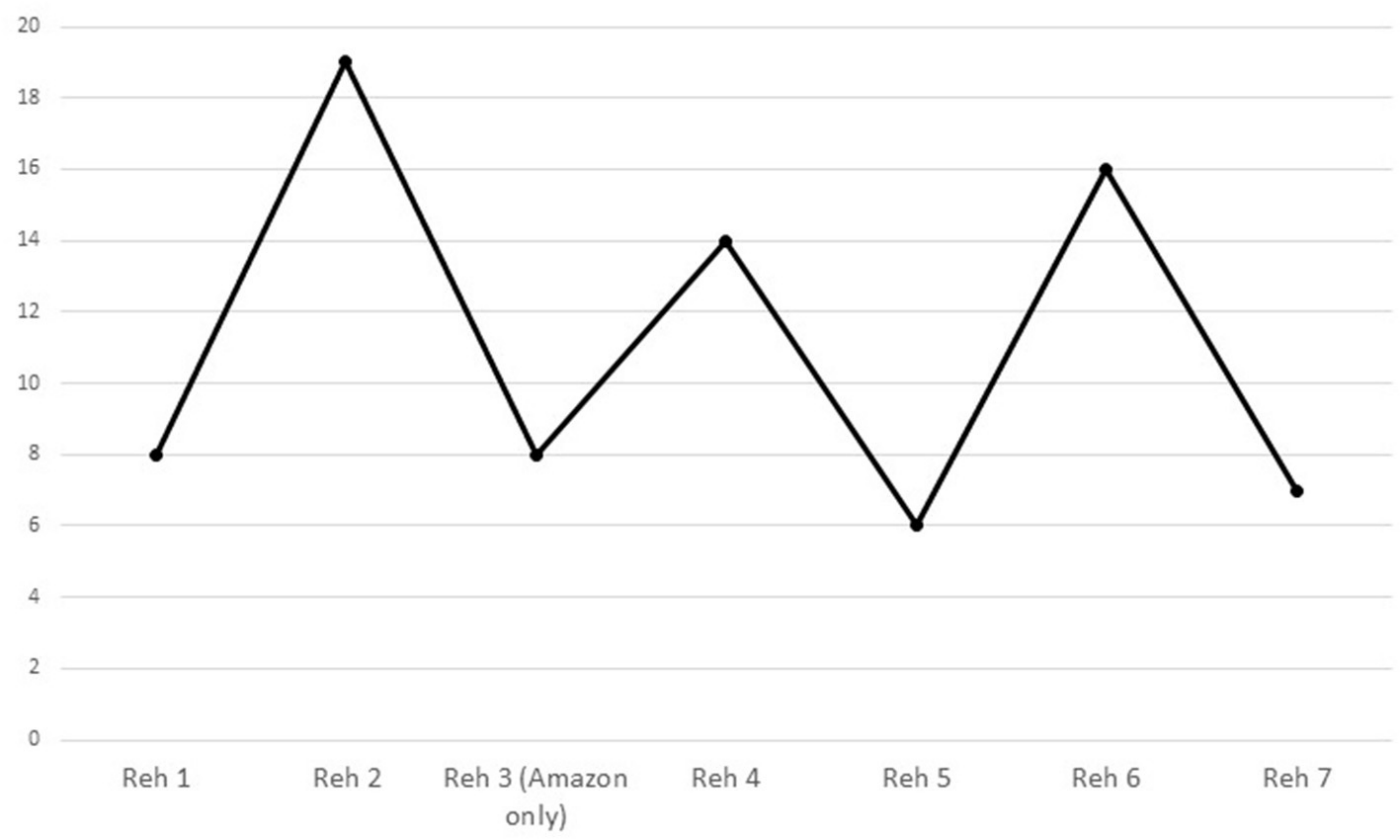
Utterances by both musicians *showing solidarity* in each rehearsal.

a) Attributable to instrument: The content of rehearsal talk differed statistically significantly between the musicians, once a Bonferroni correction had been applied, only insofar as the singer made more references to the meaning and interpretation of the lyrics of the songs in each of the rehearsals (*M* = 4.85, *SD* = 5.24) than the viola player (*M* = 0.23, *SD* = 0.44, U = 17.5, Z = −3.63, *p* < 0.001), and its nature differed only insofar as the singer was significantly more likely to make suggestions (*M* = 13.46, *SD* = 10.27) than the viola player (*M* = 3.92, *SD* = 2.25, U = 24.5, Z = −3.01, *p* = 0.002).
b) Attributable to whether or not the song was being memorized: No differences could be attributed to memorization.
c) Attributable to song: Significantly more requests for orientation were made when rehearsing “Amazon” (*M* = 2.21, *SD* = 2.46) than “Homer” (*M* = 0.17, *SD* = 0.39, U = 22, Z = −2.89, *p* = 0.004).
d) Attributable to time: The only significant change over the course of rehearsals was that, as the musicians became more familiar with each other and with the music, *Showing solidarity* [χ [6] = 14.73, *p* = 0.022] rose and fell, peaking in the second and sixth rehearsals as shown in Figure 3.

### Discussion

Almost half the rehearsal time was spent talking rather than playing and/or singing, and “Amazon” (memorized by the viola player) received marginally more rehearsal time than “Homer” (memorized by the singer), with a concomitantly larger proportion of talking. The singer initiated the majority of verbal exchanges, to a slightly greater extent in rehearsals of “Amazon” than “Homer.” The content of the musicians' talk largely concerned tempo, ensemble, entries and dynamics; the lyrics of the songs (referred to primarily by the singer); the repetition of sections; and memory. The nature of their talk was overwhelmingly positive and task-related; disagreements were rare and there was no antagonism between the musicians, who differed only in that the singer made more suggestions than the viola player. More requests for orientation were made in rehearsals of “Amazon,” most likely because it has several difficult passages for the viola that are somewhat similar but not exactly the same, and there were changes in the way the musicians expressed verbal support for each other over the course of rehearsals: showing solidarity rose in the second rehearsal, fell, and rose again as they neared their first public performances.

The shifts of focus in musicians' preparation for performance that were observed in case studies conducted by Chaffin et al. (2002) and Ginsborg et al. (2006), from basic and structural through interpretive to expressive dimensions, were not discernible in the present study. Unlike the professional duos who took part in Ginsborg and King's (2012) study, the musicians in the present study rarely asked for or gave opinions and they preferred to repeat sections rather than the whole song.

The differences between the findings of the present study and the earlier studies may, however, relate to differences between the music being learned and rehearsed, as well as differences between the 13 musicians who took part in the four studies. Gabriela Imreh (Chaffin et al., 2002) prepared the *Presto* from Bach's *Italian Concerto*, a highly structured work; Jane Ginsborg and George Nicholson prepared the first *Ricercar* from Stravinsky's *Cantata*, another highly structured work (Ginsborg et al., 2006). The four singers and four pianists recruited by Ginsborg and King (2012) all prepared the same three songs by Ivor Gurney; these were shorter, less structured and in some ways more explicitly, conventionally “expressive” than the works of Bach and Stravinsky. In that study, comparisons were made between the rehearsal strategies and socio-emotional interactions of long-established professional duos, less-established student duos, and new duos formed of one student and one professional, but the development of both strategies and familiarity was constrained by the short-term nature of their collaboration. By contrast, the singer and the viola player in the present study were not only working together on a daily basis over the course of a week, but living and working in the same environment. Furthermore, the songs by Boris Tchaikovsky were quite different from those used in previous studies. It is not surprising that their lyrics were particularly salient, as they were in Russian, a language that the singer had studied although she was not fluent in it, while it was completely unknown to the viola player. The style of the music was new to both musicians and, aside from its interpretation in the light of the meaning of the lyrics, both songs presented technical challenges, from the switches and articulation in “Amazon” and harmonics in “Homer” for the viola player, to the tongue-twister enunciation of the words in “Amazon” and the long breaths in “Homer” for the singer. In future studies it could thus be hypothesized that the ways in which verbal exchanges are patterned relate to musicians' backgrounds, the nature of their collaboration, and the repertoire to be rehearsed and performed. A great deal more research is needed, however, to investigate the influences of different variables on patterns of verbal exchange.

While the findings of this content analysis of rehearsal talk add to those of similar analyses, they represent only what can be learned about the cognitive and social processes underlying preparation for performance from the observation of what musicians talk about, and how they talk about it, when they rehearse. This first study was also designed to capture the musicians' attention to particular features of the music, categorized as musical dimensions, during the initial rehearsal period. Its findings informed Study 2, which explored the extent to which these features, represented by annotations on copies of the musicians' scores, were stored as cues for retrieval when the songs were performed from memory, both immediately after the rehearsals had been completed and again 10 months later; data from both studies were compared in Study 3.

## Study 2

### Introduction

According to Chaffin et al. (2016), successful memorization is the result of spontaneous *serial cuing*^3^ and the deliberate cultivation of *content-addressable* memory for the music. Serial cuing occurs as the musician learns to play or sing a particular piece of music, finding that each passage begins to cue the next one in terms of both motor processes and auditory imagery. Content-addressable memory, by contrast, is achieved through deliberate and thoughtful practice, described by Ericsson (2016) as “the engagement with full concentration in a training activity designed to improve a particular aspect of performance with immediate feedback, opportunities for gradual refinement by repetition and problem-solving” (p. 534). The acquisition of content-addressable memory enables the musician to start playing or singing at the beginning of a particular passage, such as “the beginning of the second verse” or “the coda.”

The results of longitudinal case studies involving the pianist Gabriela Imreh (e.g., Chaffin et al., 2002, 2003; Chaffin, 2007), the cellist Tânia Lisboa (e.g., Chaffin et al., 2010; Lisboa et al., 2018), and the singer Jane Ginsborg (e.g., Ginsborg et al., 2012) suggest that, as musicians practice independently and rehearse with others, they pay attention to particular features of the music that, like the dimensions described in Study 1, can be categorized as *structural, basic, interpretive, and expressive*. In these case studies, the musicians were asked to record their thoughts during practice and rehearsal in the form of annotations on copies of the musical score at the end of the rehearsal period (*rehearsal features*) and again when they had performed from memory. A subset of rehearsal features was found to function as cues that can be described as landmarks in the musician's mental representation of the work and that—as the result of studies in which musicians were asked to write out the score from memory, months or years later (e.g., Chaffin et al., 2002; Ginsborg and Chaffin, 2011)—have come to be known as *performance cues*, even though they cannot be shown to serve as cues for retrieval in performance until there is evidence of their doing so in repeated performances. It is assumed, however, that the features to which the musician attended during practice and rehearsal and did not recall in performance are those that have become irrelevant or have been assimilated by the musician to the point that they are performed automatically.

Until Ginsborg et al. (2012) reported on their study of the potential for spontaneous thoughts in a first performance to serve as performance cues in a second performance, drawing on the first author's own memorization and performance from memory of Schoenberg's Two Songs op. 14, musicians had not been asked about their spontaneous thoughts while performing, which might also function as performance cues in subsequent performances. Such thoughts might concern, for example, features of the music they had not noticed before; their own reactions to the music as they were performing it; and even distractions such as unwelcome and unhelpful thoughts. The following hypotheses were tested: (1) performance cues prepared during practice, indicated at the time as rehearsal features (*core performance cues*), would be retained from the first to the second performance; (2) a proportion of rehearsal features would be retained as performance cues in either one or the other but not both performances (*non-core performance cues*); and (3) a proportion of spontaneous thoughts in the first performance would recur in the second performance. In that study it had not been feasible for the musicians to give a second public performance and the solution chosen was for the singer to video-record, transcribe and analyze data from an *in vivo* reconstruction of the songs from memory, 4 months after she had last looked at them, first without and then with live piano accompaniment. The first and second hypotheses were supported, but not the third, since the data were so sparse.

#### Aim

The aim of the present study, then, was to replicate and extend the research reported by Ginsborg et al. (2012), in which the musicians were also practitioner-researchers. Each of the two musicians—both experienced professionals with long experience of rehearsing and performing with their own regular duo partners, but who did not know each other well and had never worked together before—would memorize one of the songs to be performed. On this occasion, rather than making *in vivo* reconstructions of the song from memory, they would give second and third performances, live, to audiences (see Bennett and Ginsborg, 2018). At the end of the rehearsal period they would annotate copies of their scores to indicate the features of the music to which they had attended, and after each performance they would annotate them again to indicate their thoughts while performing; these could subsequently be identified as core and non-core structural/basic, interpretive and expressive performance cues and spontaneous thoughts, and comparisons could be made between the two musicians' use of rehearsal features and performance cues in the songs, according to whether or not they were being prepared for performance from memory.

#### Research Questions

The following questions were addressed:

1. To which rehearsal features did the musicians attend individually and together, when memorizing and not memorizing?
2. To what extent did rehearsal features remain salient in each of the memorized and non-memorized performances?
3. Overall, what proportions of rehearsal features were retained in memorized performances as core and non-core performance cues?
4. What proportions of spontaneous thoughts could be considered *functional performance cues* insofar as those that occurred in Performance 1 recurred in Performance 2 and/or Performance 3, and those that occurred in Performance 2 recurred in Performance 3?

### Method

The musicians annotated copies of their scores to indicate the locations of rehearsal features (after the end of the final rehearsal) and performance cues (after each of the three performances) in the following categories and sub-categories, as shown in Table 7: *structural, basic, interpretive, expressive, memory, coordinate*, and *shared (expressive and coordinate)* (see Appendix B for excerpt from singer's annotated score of “Homer”). *Memory* and *coordinate* were additions made by the musicians to the categories of feature and performance cue used in previous research; when they annotated their scores together, the only categories they shared were *expressive* and *coordinate*.

**Table 7:** Rehearsal features and performance cues.

**Category**	**Sub-category**
Structural	Section
	Sub-section
	Switch
Basic (B)	Prepare
	Breath
	Word (B)
	Pitch
	Fingering
	Bowing
Interpretive (I)	Word (I)
	Sound
	Tempo
	Dynamics
Expressive	
Memory	
Coordinate	
Shared	Expressive
	Coordinate

### Results

#### 1. Rehearsal Features to Which the Musicians Attended Individually and Together When Memorizing and Not Memorizing

**Figure 4:**
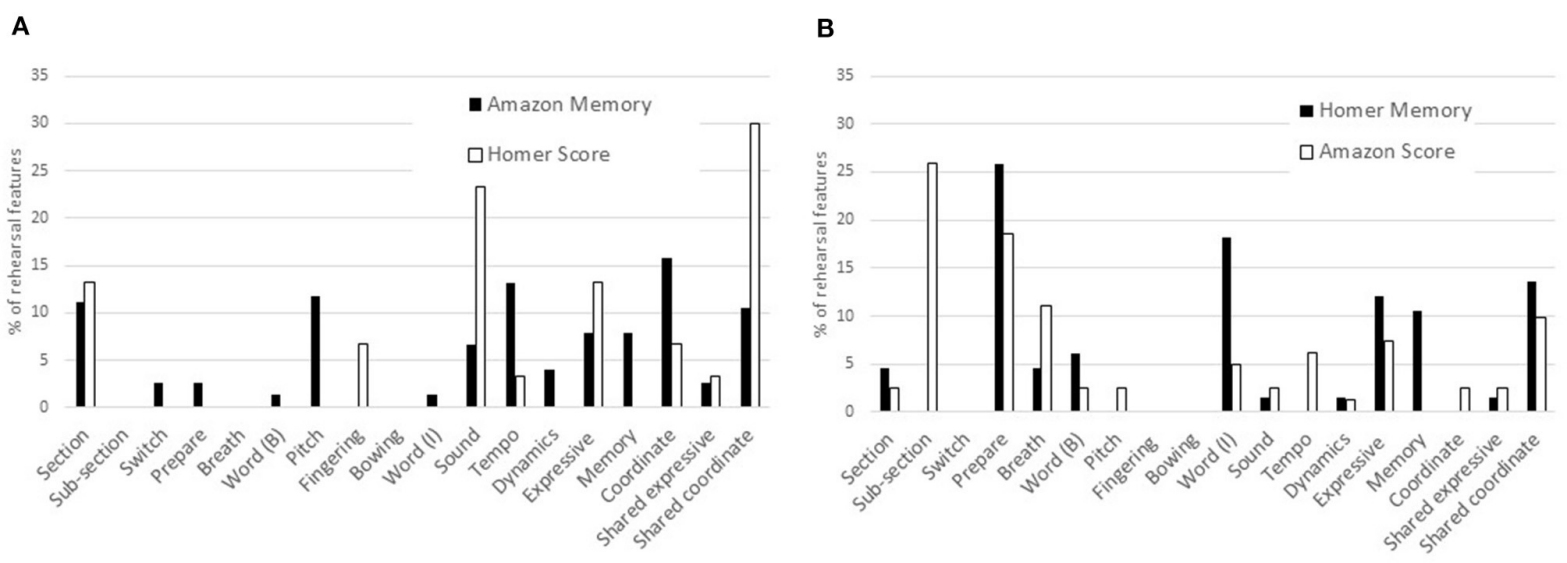
Percentages of rehearsal features in each category: viola player **(A)** and singer **(B)** memorizing and reading from the score.

The numbers of locations of rehearsal features in each category were calculated as percentages of all rehearsal features indicated by the musicians at the end of the rehearsal period. For example, the viola player noted 76 locations of rehearsal features in “Amazon” of which 11 (14.5%) referred to structural features (*section, switch*), 12 (15.8%) basic (*prepare, word, pitch*), 19 (25.0%) interpretive (*word, sound, tempo, dynamics*), etc. The full data representing numbers and percentages of rehearsal features and performance cues, which were reported after each performance, are provided in Appendix C. Figures 4A,B illustrate comparisons between the percentages of rehearsal features in each category indicated by the viola player and singer, respectively, when memorizing and not memorizing.

According to the viola player's annotations on the score of “Amazon,” which she had memorized, her most salient rehearsal features occurred at the beginnings of verses (*section*), where intonation presented potential challenges and tempo needed to be controlled, and where she needed to follow the singer. Having prepared but not memorized “Homer,” the viola player noted much larger proportions of rehearsal features relating to sound and expressivity. The proportion of *shared coordinate* rehearsal features (i.e., where the musicians took joint responsibility for the ensemble) was three times as large for “Homer” (memorized by the singer but not the viola player) as for “Amazon.”

The singer's most salient rehearsal features, according to her annotations on the score of “Homer,” which she had memorized, occurred at particular words, typically related to their meaning, at locations where she needed to be particularly expressive, and where memory presented potential challenges. Locations marked *prepare*, typically before entries where she needed to count, think, watch, and listen, were salient whether or not she was preparing to perform from memory, as was joint responsibility for ensemble. In “Amazon,” which she did not memorize, attention to sub-sections was as salient as preparation for entries in “Homer.”

**Figure 5:**
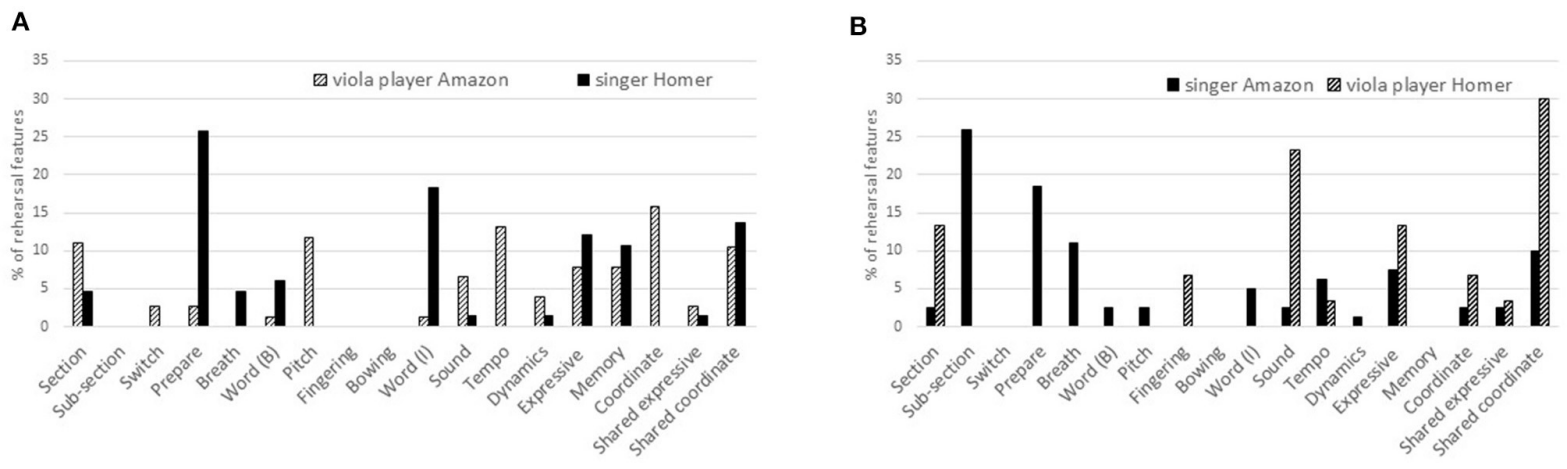
Percentages of rehearsal features in each category: both musicians memorizing **(A)** and both musicians reading from the score **(B)**.

Figures 5A,B illustrate the comparisons between the same percentages of rehearsal features in each category indicated by the two musicians when memorizing and reading from the score, respectively. According to the annotations on the two scores memorized by the musicians, it is clear that attention to sections was more important to the viola player than the singer, and that pitch, tempo, and coordination with the singer were important to the viola player while the singer did not attempt to coordinate with the viola player; by contrast, preparation for entries and the meaning of the lyrics were far more important to the singer than the viola player. Rehearsal features relating to expression, memory, and joint responsibility for ensemble were important to both musicians.

According to the annotations on the two scores that were not memorized by the musicians, the viola player attended to sections while the singer attended to sub-sections; the viola player focused to a greater extent than the singer on sound and expression, and a larger percentage of the viola player's rehearsal features reflected joint responsibility for ensemble.

#### 2. Extent to Which Rehearsal Features Remained Salient in Each of the Memorized and Non-memorized Performances

**Figure 6:**
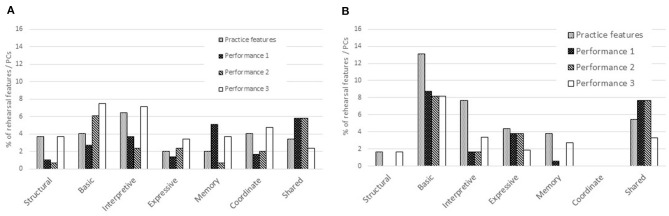
Percentages of rehearsal features that remained or became salient in performances from memory: viola player **(A)** and singer **(B)**.

Figures 6A,B illustrate the percentages of rehearsal features calculated as percentages of all annotations made by the viola player and singer, respectively, on the scores of each song, in each of the broad categories (structural, basic, interpretive, expressive, memory, coordinate, and shared) that remained or became salient in the three performances given from memory. The full data representing numbers and percentages of all annotations representing rehearsal features and performance cues are provided in Appendix D. For the viola player, who made 294 annotations on all the copies of her score of “Amazon,” structural features were much less salient in the first and second performances than the third, which took place 10 months after the initial rehearsal period; basic features were also more important in the second and third performances than the first. The pattern for proportions of features in the interpretive, expressive and coordinate categories was similar to that for features in the structural category, although there was a higher proportion of interpretive features. Memory features were most important in the first performance and to only a slightly lesser extent 10 months later; shared features were particularly salient in the first two performances. For the singer, who made 183 annotations on her scores of “Homer,” basic, interpretive, expressive, and memory performance cues can be seen to represent a sub-set of rehearsal features in the same categories, as predicted by performance cue theory, although there were more locations requiring attention to memory in rehearsal and the third performance. As already noted, other than at mutually agreed locations, the singer relied on the viola player to coordinate with her.

**Figure 7:**
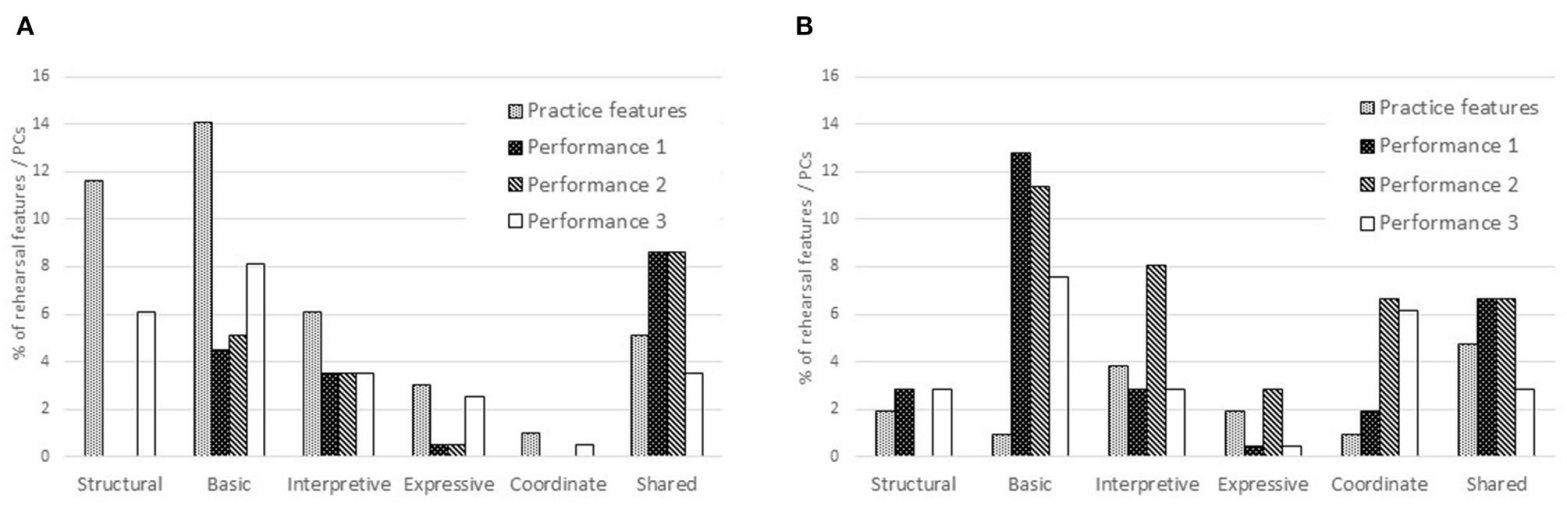
Percentages of rehearsal features that remained or became salient in performances reading from the score: singer **(A)** and viola player **(B)**.

Figures 7A,B illustrate the percentages of rehearsal features in each of the broad categories that remained salient in the three performances given by the singer and viola player, respectively, while reading from the score. For the singer, who made 198 annotations on her score of “Amazon,” structural and basic features were most salient during the rehearsal period and again 10 months later. There was a similar pattern for expressive features, while attention to interpretive features—important during rehearsal—was less prominent and remained stable throughout the three performances. By contrast, the viola player, who made 211 annotations on her score of “Homer,” indicated that basic features that had received little attention during the rehearsal period were highly salient in the first and second performances, although less so in the third; interpretive features received considerably more attention in the second than the first and third performances.

#### 3. Proportions of Rehearsal Features That Were Retained, Overall, in Memorized Performances as Core and Non-core Performance Cues

Core performance cues are defined as performance cues prepared during practice, indicated at the time as rehearsal features and indicated again after (i.e., recurring in) all subsequent performances; non-core performance cues are defined as rehearsal features recurring in one or more but not all subsequent performances. The full data representing numbers of locations at which thoughts in each category were noted to have occurred during the rehearsal period and in each of the three performances (“Amazon” for the viola player, “Homer” for the singer) are presented in Appendix E. For example, the viola player indicated major sections at nine locations after the rehearsal period and also at six of those locations in the third performance. “Section” thus represented a core performance cue for her, while “sub-section” was a spontaneous thought that occurred only in the first performance and on no other occasion, and “breath” was a spontaneous thought that occurred at five locations in the first performance and served as a cue for retrieval in the second, thus representing a non-core performance cue.

**Figure 8:**
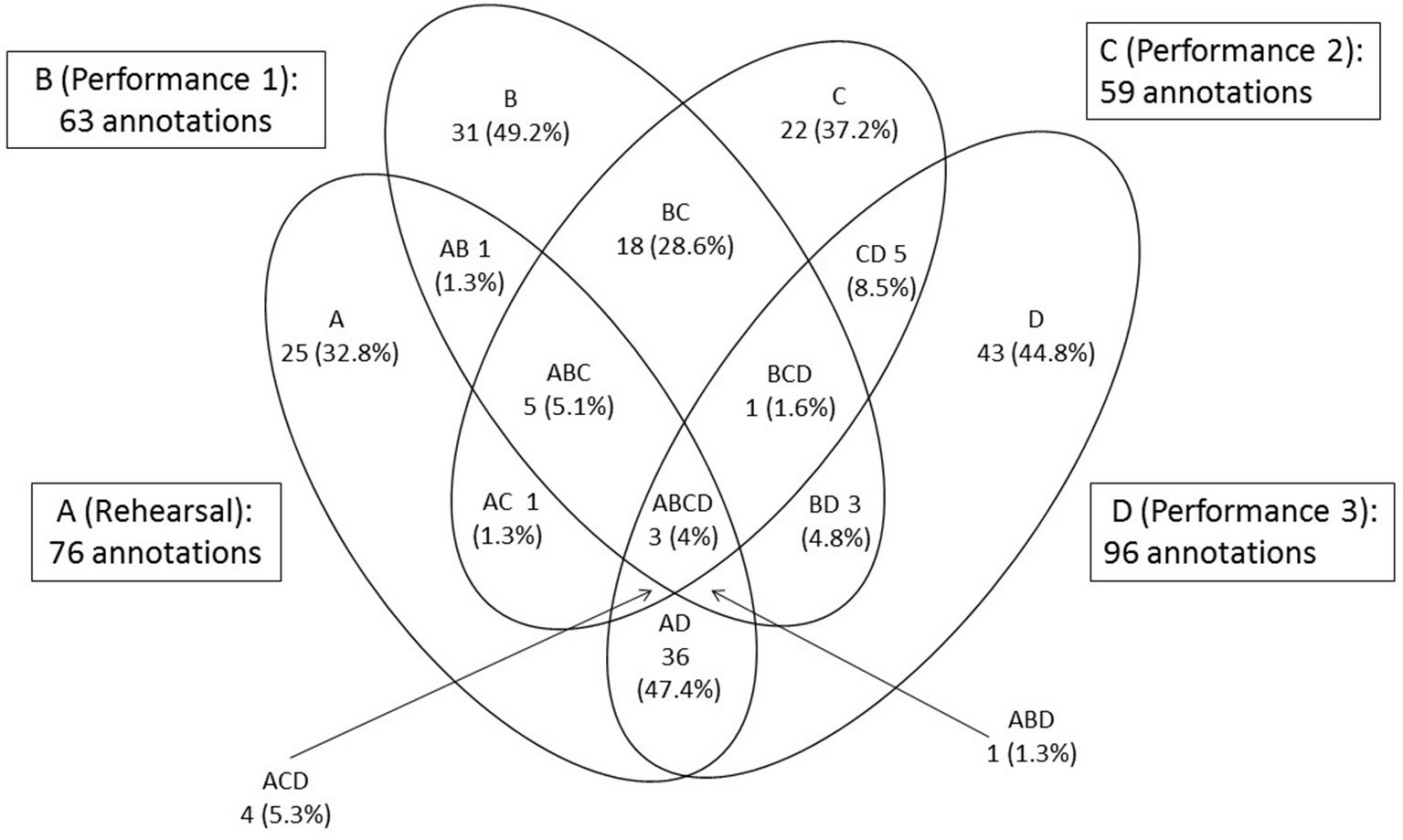
Rehearsal features and thoughts in performance from memory: viola player.

At the end of the rehearsal period the viola player made annotations on the score of “Amazon” representing 12 of the 18 categories at 76 locations; in other words, she recorded 76 rehearsal features. Of these, she recorded three in all three performances from memory; thus the percentage retained as core performance cues was 3.95%. She retained 10 rehearsal features in two of the three performances and 38 in one performance only (36 in the third, which took place 10 months after the initial rehearsal period); the percentage retained as non-core performance cues was thus 63.2%. Figure 8 illustrates the numbers of rehearsal features and thoughts in performance. Thoughts in each performance are shown as percentages of rehearsal features or their first spontaneous occurrence: 294 annotations made in total. These thoughts are identified in **Figure 10** as core and non-core performance cues and spontaneous thoughts.

**Figure 9:**
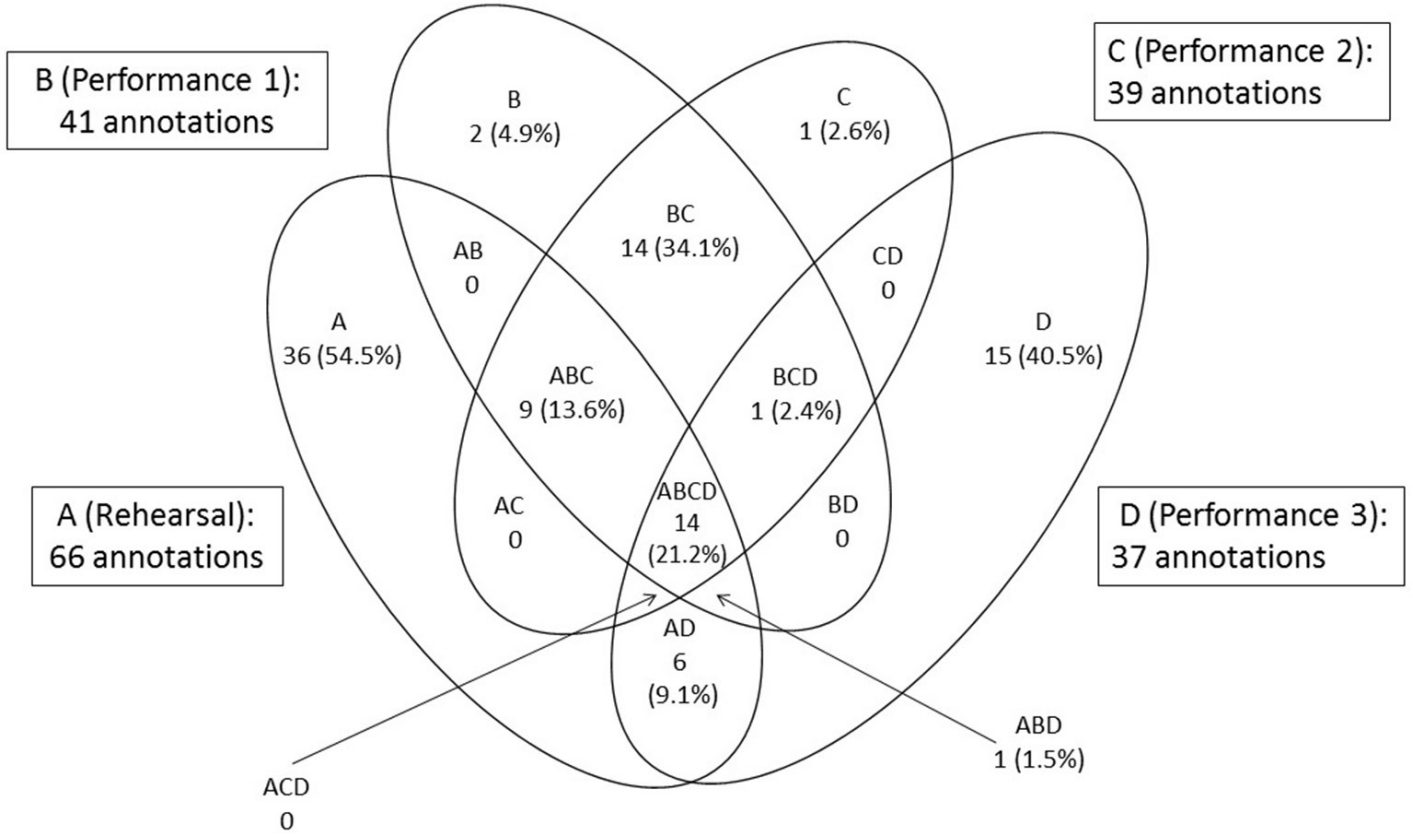
Rehearsal features and thoughts in performance from memory: singer.

**Figure 10:**
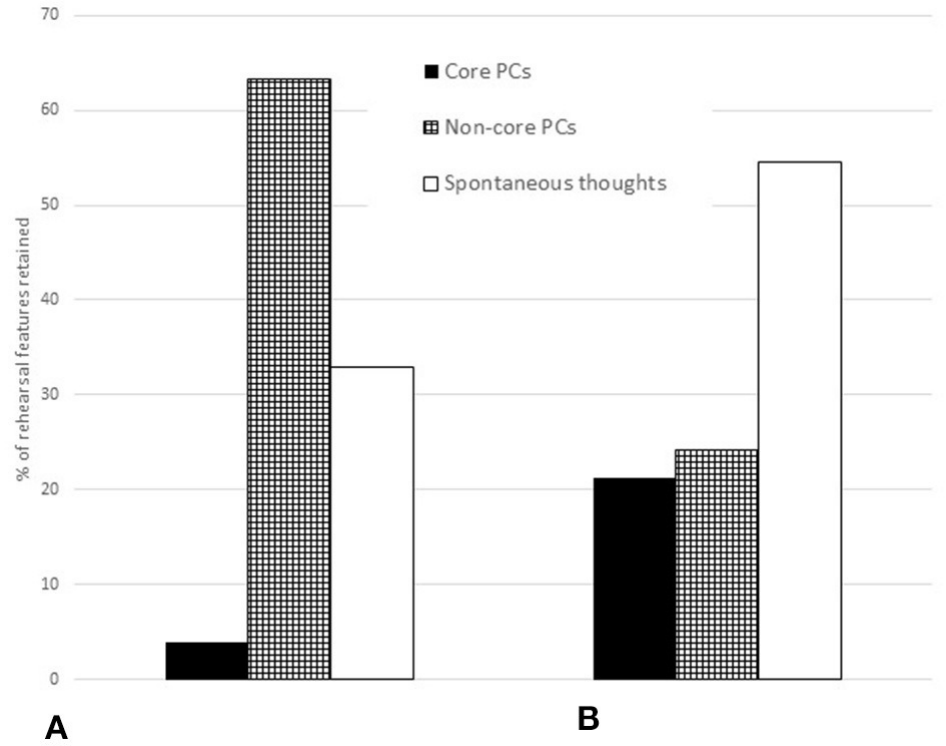
Percentages of rehearsal features retained by the musicians as core and non-core performance cues and spontaneous thoughts. **(A)** Viola player and **(B)** Singer.

The singer recorded 66 rehearsal features in eight of the 16 categories relevant to her. Of these she retained 14 in all three performances from memory, or 21.2%. She also retained 10 rehearsal features in two of the three performances and six in one performance only (again, the third); the percentage retained as non-core performance cues was thus 24.2%. Figure 9 illustrates the numbers of rehearsal features and thoughts in performance. Again, thoughts in each performance are shown as percentages of rehearsal features or their first spontaneous occurrence: 183 annotations made in total. These thoughts, too, are identified in Figure 10 as core and non-core performance cues and spontaneous thoughts.

The comparison between the percentage of rehearsal features retained by the viola player and singer, respectively, as core and non-core performance cues is illustrated in Figure 10.

#### 4. Proportions of Spontaneous Thoughts That Could Be Considered Functional Performance Cues

After Performance 1, the viola player made 31 annotations on the score in 11 categories, at previously unmarked locations; in other words, she recorded 31 spontaneous thoughts. Of these, one was retained as a functional performance cue in both subsequent performances (3.2%) while 18 were retained as functional performance cues in Performance 2 (58.1%) and three in Performance 3 (9.6%). After Performance 2, the viola player recorded 22 spontaneous thoughts of which five were retained as functional performance cues in Performance 3 (22.7%).

The singer recorded 17 spontaneous thoughts. Of these, one was retained as a functional performance cue in both subsequent performances (5.8%) and 14 were retained as functional performance cues in Performance 2 only (82.4%). In that performance she recorded one further spontaneous thought, which was not retained in Performance 3.

**Figure 11:**
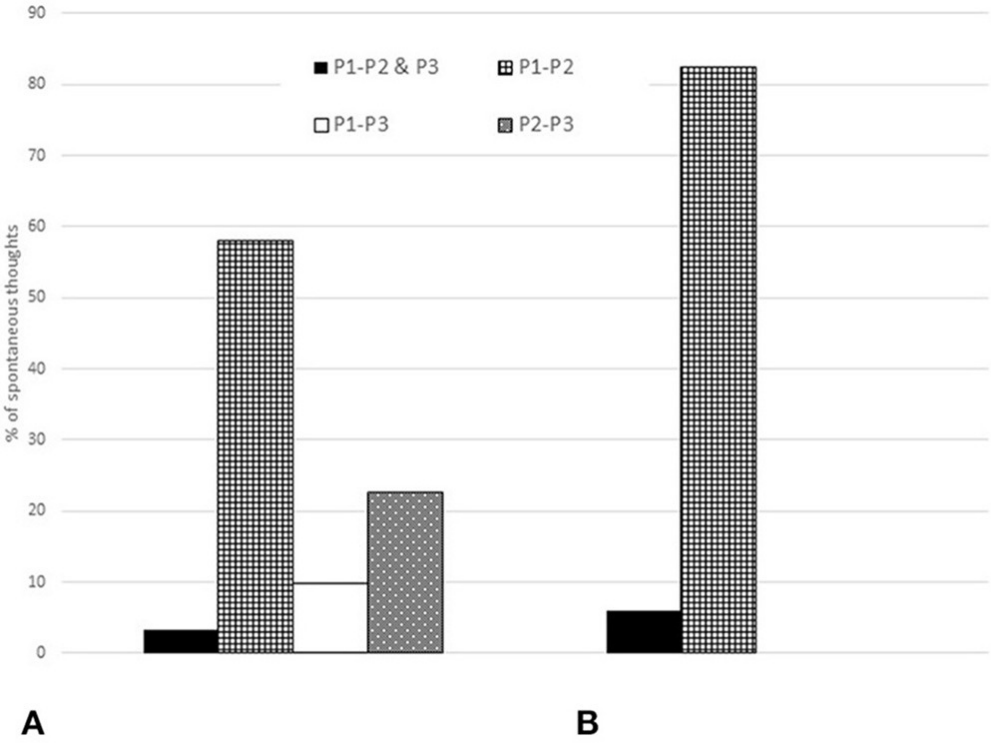
Percentages of spontaneous thoughts retained as functional performance cues. **(A)** Viola player and **(B)** Singer.

The comparison between the percentages of spontaneous thoughts retained by the viola player and singer, respectively, as functional performance cues is illustrated in Figure 11.

### Discussion

In order to consider the extent to which performance cues are prepared in the course of practice and rehearsal, and thus to explore the role of spontaneous thoughts, we began by comparing the rehearsal features identified by the singer and viola player when they were preparing to perform from memory and when they were reading from the score (research question 1). Certain categories of rehearsal feature were more salient for the musicians when memorizing (e.g., for the viola player: pitch, tempo, coordination with singer; for the singer: prepare, and the meaning of the lyrics); by contrast, when they were reading from the score, they could afford to focus on other categories of feature (e.g., sound for the viola player, subsection boundaries for the singer).

We went on to investigate the extent to which the different categories of rehearsal feature remained salient in each of the memorized and non-memorized performances (research question 2). For the viola player, memory was most salient in the first and, to a lesser extent, third performances from memory, while basic features were most salient in the second and third performances, and interpretive features and co-ordinate with the singer were most salient in the third performance from memory. For the singer, basic features were highly and equally salient in all three performances from memory, as were shared performances for both musicians in the first and second performances, although less so in the third.

To test the theory that performance cues represent the subset of rehearsal features that are not forgotten, assimilated or automated (Chaffin et al., 2002), we considered (research question 3) the proportions of rehearsal features that were retained in all three performances from memory (core performance cues) and one or more but not all three (non-core performance cues), and found that the viola player retained 3.95% as the former and 63.2% as the latter: a total of 67.15%. By contrast, the singer retained 21.2% of rehearsal features as core performance cues and 24.2% as non-core performance cues, a total of 45.4%. Taken together, these findings indicate that attention to rehearsal features does underlie retrieval from memory, as suggested by performance cue theory, but that spontaneous thoughts while performing can also play an important role, as suggested by Ginsborg et al. (2012).

Accordingly, we explored (research question 4) the proportions of spontaneous thoughts that recurred in subsequent performances. For both musicians, the proportions of spontaneous thoughts in the first performance that recurred in both the second and third were very small (3.2 and 5.8%, respectively). By contrast, the proportions of spontaneous thoughts in the first performance that recurred in the second were comparatively high (58.1 and 82.4%). Yet while 22.7% of the viola player's spontaneous thoughts in the second performance recurred in the third, the singer had a single spontaneous thought in the second performance that did not recur in the third. It is perhaps not surprising that relatively few spontaneous thoughts in the first—and to a lesser extent, the second—performance functioned as retrieval cues in the third, since the two performances were separated by 10 months. That the proportions of spontaneous thoughts in the first performance recurred in the second performance, however, highlights what every musician knows from experience: what happens in performance is not necessarily the same as what happens in rehearsal, and new insights can inform subsequent performances.

Overall, the findings support those of the earlier study examining the roles of spontaneous thoughts, as well as rehearsal features, in the development of performance cues. They do so, however, on the basis of data gathered in the context of live performances from memory given by the first author—who already had experience of conducting such research on her own memorization and performance—with another expert musician. Some of the differences between the results for the singer and viola player, respectively, are attributable to the different constraints on the voice and the viola as instruments. Some can be attributed to the differences between the characteristics of the two songs they memorized and performed and others to the differences between the two musicians' approaches to the task, according to their personalities and long experience as performers. Such differences are difficult to control for, and it could, of course, be argued that it is not necessary in this kind of real-world research to attempt to control for them, but they could nevertheless inform hypotheses to be investigated in future research.

While the findings of this analysis add to those of other longitudinal case studies investigating the development of musicians' individual and shared performance cues, and complement the findings of the content analysis of rehearsal talk reported in Study 1, further analysis was required to establish the extent to which rehearsal talk reflects attention to rehearsal features and can be inferred to predict the use of performance cues.

## Study 3

### Introduction

In Study 1 our focus of attention was the content and nature of the verbal utterances made by the members of a newly formed voice and viola duo—their talk—as they rehearsed two songs that were initially unfamiliar to them. Bales' Interaction Process Analysis was used to explore the socio-emotional and task-related nature of the talk, and a framework derived from several earlier studies (e.g., Chaffin et al., 2002; Ginsborg et al., 2006) was used to explore its content, in terms of codes representing the musicians' references to the structural/basic, interpretive, expressive dimensions of the music and strategic dimensions of the rehearsal (combined into the overarching category of *musical dimensions*). In Study 2, by comparison, our focus was the musicians' development and use of performance cues, and the data consisted in annotations on multiple copies of the musicians' scores representing structural, basic, interpretive, expressive and shared rehearsal features and thoughts while performing, some but not all of which, as we have seen, function as core and non-core performance cues (combined into the overarching category of *features and thoughts*). To triangulate these different types of data in Study 3—both of which could be categorized broadly in terms of *structure, basic, interpretive, expressive* and *memory (strategy)*—it was necessary to combine the two frameworks of 27 dimensions and 14 types of rehearsal feature and thought, respectively, so as to carry out a correlational analysis. We did not include the Bales framework for analyzing socio-emotional interactions, since this was not relevant to the comparison of verbal data reflecting musical dimensions with documentary data reflecting musicians' thoughts while rehearsing and performing. The findings of the analysis are discussed with reference to examples taken from rehearsal transcripts.

### Material and Methods

Table 8 presents the five broad categories of type of dimension, feature, and thought; the original framework of 27 dimensions and their codes; and the 14 types of rehearsal feature and thought. Those in bold type were included in a smaller framework, constructed by omitting the dimensions that did not have counterpart features and/or thoughts and combining dimensions where appropriate (e.g., subsuming *rubato* into interpretive *tempo*). This framework, consisting of the five broad categories divided into 13 dimensions and the features and thoughts analyzed in the present study, is shown in Table 9.

**Table 8:** Frameworks used in original analyses of rehearsal talk and performance cues.

**Type of dimension/rehearsal feature/thought**	**Dimensions (rehearsal talk)**	**Code**	**Features and/or thoughts (annotations)**
Structure	**Structure**	**BSt**	**Major sections/sub-sections**
	**Switch**	**BSw**	**Switch**
Basic	Articulation	BA	
	**Breath**	**BB**	**Breath**
	Composition	BC	
	Dynamics	BD	
	**Ensemble**	**BEns**	**Co-ordinate/shared co-ordinate**
	**Entries**	**BEnt**	**Prepare**
	Harmony	BH	
	Meter	BM	
	Notation	BN	
	**Pitch**	**BP**	**Pitch**
	Technique	BTec	
	Tempo	BTem	
	The instrument/voice	BI	
	**Words**	**BW**	**Word (Basic)**
Interpretive	Articulation	IA	
	**Color**	**ICol**	**Sound**
	**Dynamics**	**ID**	**Dynamics**
	Harmony	IH	
	Meter	IM	
	Rubato	IR	equivalent to Tempo (interpretive)
	Phrasing	IP	
	**Tempo**	**IT**	**Tempo**
	**Words**	**IW**	**Word (Interpretive)**
Expressive	**Expressive**	**E**	**Expressive**
Memory (strategy)	**Memory**	**SM**	**Memory**

**Table 9:** Framework of dimensions, features and thoughts used in present study.

**Type of dimension/feature/thought**	**Dimensions (rehearsal talk)**	**Code**	**Features and/or thoughts (annotations)**
Structure	Structure	BSt	Major sections/sub-sections
	Switch	BSw	Switch
Basic	Breath	BB	Breath
	Ensemble	BEns	Co-ordinate/shared co-ordinate
	Entries	BEnt	Prepare
	Pitch	BP	Pitch
	Words	BW	Word (Basic)
Interpretive	Color	ICol	Sound
	Dynamics	ID	Dynamics
	Rubato/Tempo	IR/T	Tempo
	Words	IW	Word (Interpretive)
Expressive	Expressive	E	Expressive/shared expressive
Memory (strategy)	Memory	SM	Memory

#### Analyses

Four sets of Pearson correlations were calculated, one for each musician rehearsing and performing each song. Five variables were correlated: utterances made while rehearsing the two songs, and annotations representing rehearsal features and thoughts in each of the three performances. The data (shown in full in Appendix F) were the numbers of utterances and annotations in each of the 13 sub-categories shown in Table 9, expressed as percentages of all the coded utterances made by the musician while rehearsing the song in question, and all the features and thoughts recorded by the musician after the rehearsal period and each of the performances.

### Results

#### Viola Player: “Amazon” (Memory)

There were no significant correlations between utterances and features or thoughts in the first, second or third performance, while there were significant correlations between features and thoughts in each of the first (*r* = 0.572, *p* = 0.041), second (*r* = 0.728, *p* = 0.005) and third performances (*r* = 0.803, *p* = 0.001); between thoughts in the first and each of the second (*r* = 0.636, *p* = 0.019) and third performances (*r* = 0.796, *p* = 0.001); and between thoughts in the second and third performances (*r* = 0.654, *p* = 0.015).

#### Singer: “Amazon” (Reading From the Score)

There were no significant correlations between utterances and features or thoughts in the first, second or third performance, although there were significant correlations between features and thoughts in the third performance (*r* = 0.943, *p* < 0.001) and between thoughts in the first and second performances (*r* = 0.997, *p* < 0.001).

#### Singer: “Homer” (Memory)

There were significant correlations between utterances and thoughts in the third performance (*r* = 0.581, *p* = 0.037) and between features and thoughts in each of the first (*r* = 0.771, *p* = 0.002), second (*r* = 0.745, *p* = 0.003) and third performances (*r* = 0.890, *p* < 0.001); between thoughts in the first and each of the second (*r* = 0.997, *p* < 0.001) and third performances (*r* = 0.695, *p* = 0.008); and between thoughts in the second and third performances (*r* = 0.655, *p* = 0.015).

#### Viola Player: “Homer” (Reading From the Score)

Similarly, there were significant correlations between utterances and thoughts in the third performance (*r* = 0.642, *p* = 0.018) and between features and thoughts in each of the first (*r* = 0.768, *p* = 0.002), second (*r* = 0.941, *p* < 0.001) and third performances (*r* = 0.839, *p* < 0.001); between thoughts in the first and each of the second (*r* = 0.725, *p* < 0.005) and third performances (*r* = 0.713, *p* = 0.006); and between thoughts in the second and third performances (*r* = 0.783, *p* = 0.002).

### Discussion

We asked if rehearsal talk reflects attention to rehearsal features and predicts the use of performance cues by attempting to correlate the dimensions to which the musicians' talk referred, the features they noted after rehearsing, and the thoughts they had while performing, using the same sub-categories within the broader structural, basic, interpretive, expressive, and memory categories. Correlations between features and thoughts in performance were almost all significant (the exception was that significant correlations for the singer were only between thoughts in the first and second performances, and between attention to features and the third performance of “Amazon,” the song she did not memorize) and support the findings of Study 2. Correlations between rehearsal talk and the other variables, however, were asymmetrical: for the singer, rehearsal talk predicted thoughts in the third performance of “Homer” from memory (but not attention to features, or either of the first two performances) while for the viola player, rehearsal talk predicted thoughts in the third performance of same song, which she did not perform from memory. Somewhat surprisingly, rehearsal talk did not reflect attention to rehearsal features, nor thoughts in the performances that took place on the same day as the final rehearsal. That it predicted both musicians' thoughts in the performance of “Homer” that took place 10 months later, but not “Amazon,” suggests that the apparent asymmetry between the findings for the singer and viola player, respectively, arose from differences between songs and the musicians' approach to it, rather than whether or not the musician was performing from memory.

**Figure 12:**
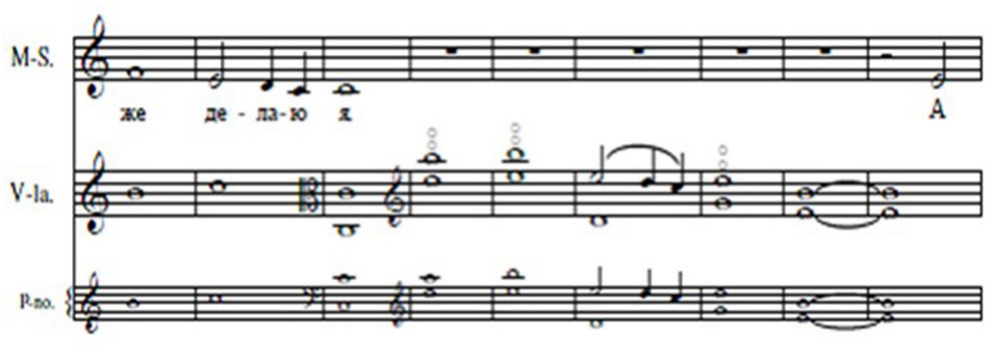
“Homer” bars 29–37.

This can most effectively be illustrated by reference to the transcripts of the musicians' utterances and their annotations. For example, see bars 29–37 of “Homer” (Figure 12), the closing bars of the first verse of the song.

In Kipling's original, the text of the whole of the first verse reads as follows:

When 'Omer smote 'is bloomin' lyre,

He'd 'eard men sing by land an' sea;

An' what he thought 'e might require,

'E went an' took – the same as me!

The Russian text corresponding to “the same as me!” is “[и так] же делаю я,” pronounced “[ee tak] zhe dyelyayoo ya.” Literally, it means “and so do I.”

The musicians rehearsed the song on six occasions. In the first two rehearsals they sang and played through the passage twice, and the singer spoke and translated the words aloud, but they did not discuss the text, its setting or how they were performing it. In the third rehearsal they sang and played the passage four times, focusing on the harmonics for the viola in the fourth, fifth and seventh bars:

Viola player: Just going to lead in with these wretched harmonics. So it is… last bar of first verse (humming while playing) *[This utterance was coded* BEn**s** –* co-ordinate/shared co-ordinate]*

Viola player: He! Okay! We're both hungry <*laugh*>

[Viola player started played from the beginning of the third bar and both musicians continued to the end of the song.]

Viola player: Did I halve the speed?

Singer: You did. <*laugh*>

Viola player: I learned it wrong.

Singer: It sounded nice.

In the fourth, fifth and sixth rehearsals the musicians sang and played through the passage seven times in all, without comment, but at the end of the sixth rehearsal the following dialogue took place:

Singer: Now I realized that when I was singing about “thus do I,” I actually am [the character in the song is] being a bit embarrassed about that. You know, when he's, right at the beginning

Viola player: Right, yeah <*laugh*>

Singer: When he says…

Viola player: <*humming something*>

Singer: Yes, and I do too, feel a bit embarrassed about it. And then, but, they listened quietly. But… you're listening quietly. And then I realized that if I indicate the winking, 'cause I can't wink with both eyes but I can wink with my right eye… And I know it's hamming it up, and I don't altogether approve of that but… I think in fact, in a little way, it's quite helpful

Viola player: I think it's great

Singer: All right, should we leave it there and do the annotations?

**Figure 13:**
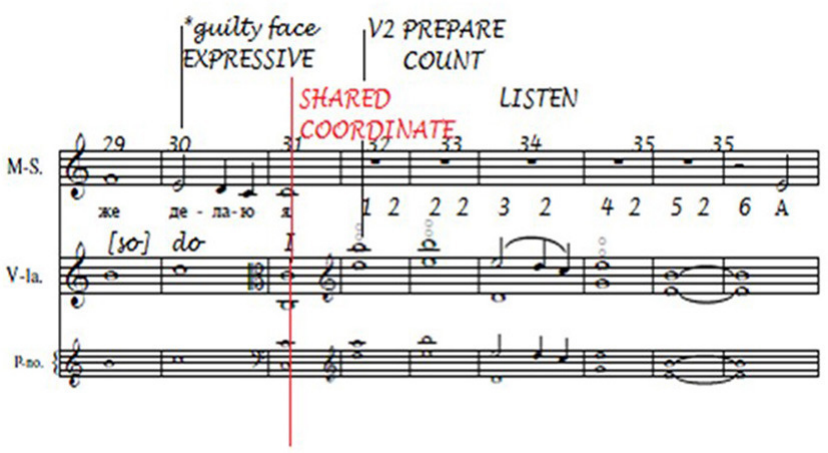
Singer's rehearsal features (“Homer”).

The annotations made by the singer on these bars of the song are shown in Figure 13 and represent the rehearsal features to which she paid attention:

Note that the expressive feature “guilty face” corresponds with the embarrassment the singer wished to convey at the end of this verse (the winking refers to the last verse of the song; see Appendix A for the original text by Kipling and Appendix B for the literal translation). She also noted the beginning of Verse 2 at bar 32, and it is likely that the reminder to count and listen reflected the viola player's halving of the speed referred to in the transcript. “Shared coordinate” is in red because she and the viola player agreed that they had both taken responsibility for coordinating at this location.

**Figure 14:**
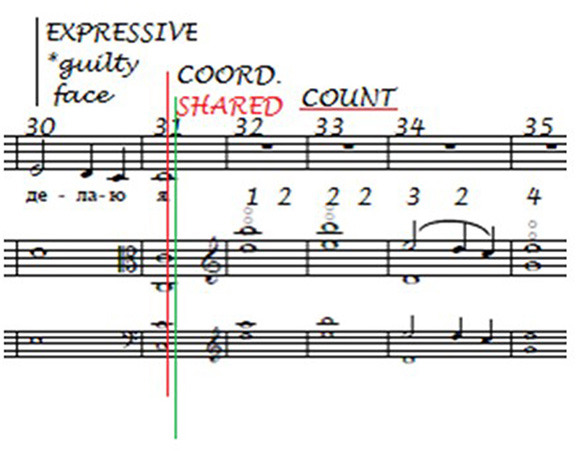
Singer's thoughts in performances 1 and 2 (“Homer”).

As shown in Figure 14, the annotations made on a clean copy of the score after the first performance (in black) and the second performance (in red; the green line indicates that the viola player agreed that this was the location of a shared performance cue), “Expressive (*guilty face*),” “Shared coordinate” and “Count” were retained in the first performance, while only the reminders to share equal responsibility for shared coordination and to count (indicated by the red underlining) were retained in the second.

**Figure 15:**
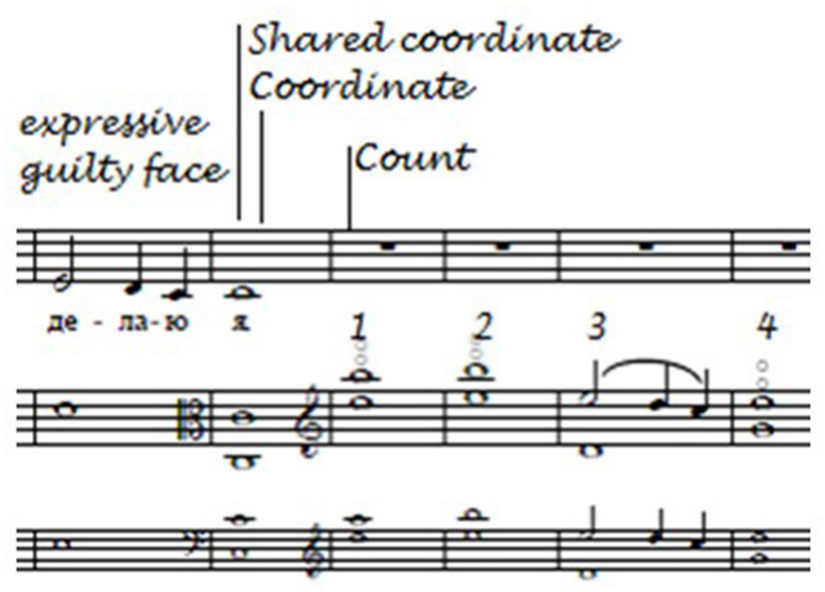
Singer's thoughts in performance 3 (“Homer”).

Once again, as shown in Figure 15, “expressive” and “guilty face” were retained in the third performance, 10 months later, as were “shared coordinate” and “count” (these two representing core performance cues), while “coordinate,” a spontaneous thought in the first performance, was retained in both the second and third performances.

What musicians say in rehearsal can thus be seen to have little connection with what they do in the context of rehearsal or a performance. While it may seem counter-intuitive for the musicians to have rehearsed this passage six times but to have discussed it only twice (first in relation to the viola player's preoccupations with the harmonics and the speed, second in relation to conveying the meaning of the text via extra-musical means), the purpose of music making is to play or sing, not to talk, and the findings do in fact confirm those of previous research. Ginsborg and King (2012), for example, found in their observations of single rehearsals carried out by student and professional singer-pianist duos that the latter talked less and played and sang more than the students, and the professional musicians who had worked with their own duo partners for more than 10 years talked less than they did when they were working either with a new professional partner or a student partner for the first time.

## General Discussion

In this study the two authors, both expert and formerly professional musicians, fulfilling the roles of practitioner-researchers, came together to form a new duo for the purposes of undertaking a longitudinal case study in which they would track their preparation for performance. It provided the opportunity, first, to replicate and extend two previous studies of verbal communication between musicians. The first was Ginsborg et al.'s (2006) content analysis of the rehearsal talk of a singer-pianist duo who had been working together, by the time the data were collected, for 40 years, and had been living together as a couple for nearly as long. In order to explore the role of familiarity, metaphorical as well as literal, on the interactions of duo partners, Ginsborg and King (2012) compared the rehearsal talk of established singer-pianist duos, including a married couple, with that of newly formed duos, albeit in single rehearsals rather than the month-long rehearsal period of the earlier investigation. Study 1, in the present article, focused on the development of social familiarity between the two musicians, who had met each other as academics, but only relatively infrequently as they live and work in the UK and Australia, respectively; the research could only be undertaken because the first author was visiting Australia for the purposes of a study visit and staying with the second author for a week. The frameworks for analysis used in those two studies were applied in the present study, adapted appropriately to a singer and viola player, and produced findings that both confirmed the results of the earlier studies and challenged them.

For example, while their verbal interactions were positive and task-related, as were those of the musicians who took part in the earlier studies, they spent more time talking than might have been expected on the basis of the findings of Ginsborg and King (2012). This could, however, be attributable to the nature of the songs they were preparing for performance. The singer and pianist in Ginsborg et al.'s (2006) study preparing the first Ricercar from Stravinsky's *Cantata* both knew the music of the composer and the specific piece before they began working on it; although the lyrics are in archaic English they were still relatively comprehensible to the musicians on first sight. Although the songs prepared in Ginsborg and King's (2012) were new to the participants, they had all performed other songs by the same composer, Ivor Gurney (1890–1937). In the present study, the musicians had agreed that the second author would identify a piece for singer and viola player—a comparatively rare combination—and that they would look at it for the first time together. Neither had come across the composer or his work before, and the fact that the lyrics were in Russian, and bore little resemblance to the original verses by Rudyard Kipling, could have proved an insurmountable stumbling-block had the singer not already performed many settings of Russian texts, in Russian, and if she had not been able to identify a willing translator who was prepared to produce a word-for-word translation including explanations of the meaning of Russian phrases such as “have a beard” (i.e., to be untrue). In short, the musicians talked a great deal because there was a great deal to talk about.

Study 2, in the present article, also gave the researchers the opportunity to investigate their thoughts while rehearsing and performing, as recorded in the form of annotations representing rehearsal features and performance cues. Ginsborg et al. (2012) explored the role of spontaneity in performance by asking if *all* thoughts in performance derive from thoughts while performing, or if spontaneous thoughts in one performance might provide additional insights that could only be gained in performance, rather than rehearsal. Given that it had not been feasible in that study to address the question by gathering data from multiple performances, but only from an *in vivo* reconstruction as described above, Study 2 was designed to include two performances on the same day and a third 10 months later, when the viola player was visiting the UK.

Although pianists are expected to perform solo repertoire from memory and singers to perform accompanied repertoire from memory, it is very unusual indeed for both musicians in a singer-pianist duo to perform together from memory. The singer and viola player in the present study, however, agreed that each of them would memorize and perform one of the two songs from memory, accompanying the other musician in the other. This would make it possible to compare the two musicians' thoughts while rehearsing and performing from memory and while reading from the score. It is difficult to know to what extent the findings of the comparisons reflect differences attributable to memorizing or not memorizing, as the musicians memorized different songs and those songs had quite different characteristics. In “Amazon” the syllables of the words had to be sung fast above a series of arpeggiated chords in the viola part, sometimes legato and sometimes pizzicato, including several switches; in “Homer” the viola part consisted of long held notes, many of which were harmonics, with a more expressive, long-breathed, narrative vocal line.

The key findings, however, relate to the potential for spontaneous thoughts to function as core and non-core performance cues, and the data for both the singer and the viola player show that this is indeed the case. The differences between them could once again be attributable to the differences between the songs but also to the differences between the musicians. This was the third study in which the singer had tracked and analyzed her own rehearsal and performance using similar methods; the viola player and her regular duo partner, the pianist Diana Blom, had also investigated the development of their collaborative understanding of a new piece of music the year before the present study was undertaken, but using different methods (Blom and Bennett, 2013).

Finally, Study 3, in the present article, attempted to triangulate the first two studies by investigating the extent to which the findings of the first correlated with the findings of the second, and therefore the extent to which it could be inferred that verbal communication during rehearsal reflected thoughts about the music and its performance while rehearsing, as recorded in the form of rehearsal features at the end of the rehearsal period, and thoughts while performing. We concluded that talk and action are not necessarily related to each other and thus that the study of musicians' verbal interactions is less valuable than might have been thought for determining the cognitive processes underlying preparation for performance, even though it may have its uses for exploring social processes.

The three studies, taken together, share the characteristics of all similar longitudinal case studies: findings depend on the musician(s) who undertake them, the music they prepare for performance, the context(s) in which the performance(s) take place—in this case the first two performances were relatively informal private concerts given to a small audience consisting of family and friends, while the third performance was more formal and given to a larger audience—and their understanding of the task, whether this is to articulate rehearsal processes in words or make annotations reflecting rehearsal features and potential performance cues according to an existing framework for analysis. From certain perspectives these could be seen as limitations, since they preclude generalization to other musicians, repertoire and performance contexts.

Nevertheless, the study fulfilled some valuable functions. It examined the social and cognitive rehearsal processes of a newly formed duo, as revealed by a content analysis of their talk. It addressed questions as to the role of spontaneous thoughts while performing, besides rehearsal features, in the development of performance cues. And it showed that rehearsal talk, rehearsal features and performance cues cannot usefully be combined in a single analysis. Above all, it provides an in-depth, detailed analysis of two musicians “in the real world” preparing a new work for performance together, and will—we hope—inspire others to formulate new questions and answer them in new ways.

## Data Availability Statement

The datasets generated and analyzed for this study can be made available by the first author to any suitably-qualified researcher on request.

## Ethics Statement

Ethical review and approval were not required for the study, in accordance with local legislation and institutional requirements at the time the research was carried out. Written informed consent from the participants was not required for them to participate in this study in accordance with the national legislation and the institutional requirements.

## Author Contributions

JG and DB contributed to the design of the study and conducted it together as practitioner-researchers. Preliminary analyses were carried out by both authors and JG undertook the final analyses. JG drafted the manuscript. Both authors edited the manuscript, read it and approved the submitted version.

## Conflict of Interest

The authors declare that the research was conducted in the absence of any commercial or financial relationships that could be construed as a potential conflict of interest.

## Footnotes

^1^Leonard Bernstein famously issued a disclaimer before conducting Brahms' Piano Concerto in D minor with Glenn Gould and the New York Philharmonic Orchestra in April 1962, so vehemently did he disagree with the pianist's interpretation of the work (for more information see https://web.archive.org/web/20001031125032/; http://www.rci.rutgers.edu/~mwatts/glenn/lennie.html).

^2^All three studies were reported at the International Symposium on Performance Science, in 2017, 2015 and 2019 respectively. Brief accounts of Study 1 can be found in Bennett and Ginsborg (2017) and Ginsborg and Bennett (in press), while Study 2 is outlined in Ginsborg (2020). Rehearsal features and performance cues are also discussed in Ginsborg (in press).

^3^This term replaces *associative chaining*, used by Chaffin et al. (2016). It derives from a particular explanation for serial recall based on item-to-item associations (see Lindsey and Logan, 2019) and has been now been rejected by Chaffin and his colleagues in favor of *serial cuing*, which occurs on multiple time scales.

## Supplementary Material

Supplementary Data Sheet 1 - Appendix A: Original verses by Rudyard Kipling. - Appendix B: Page of singer's annotated score. - Appendix C: Rehearsal features and performance cues (percentages represent numbers/rehearsal or performance). - Appendix D: Rehearsal features and performance cues (percentages represent numbers/all annotations per song). - Appendix E: Numbers of annotations. - Appendix F: Numbers and percentages of utterances (rehearsal talk), features, and thoughts in Performances 1, 2, and 3.
