# Supplementary material for: Developing Familiarity in a New Duo: Rehearsal Talk and Performance Cues
Source: Front Psychol. 2021 Mar 16;12:590987. doi: 10.3389/fpsyg.2021.590987 (PMC8020115; doi:10.3389/fpsyg.2021.590987)
Supplement: Supplementary Data Sheet 1 — - Appendix A: Original verses by Rudyard Kipling. - Appendix B: Page of singer's annotated score. - Appendix C: Rehearsal features and performance cues (percentages represent numbers/rehearsal or performance). - Appendix D: Rehearsal features and performance cues (percentages represent numbers/all annotations per song). - Appendix E: Numbers of annotations. - Appendix F: Numbers and percentages of utterances (rehearsal talk), features, and thoughts in Performances 1, 2, and 3. [file Data_Sheet_1.pdf]

Supplementary Materials: Appendices A - F

Appendix A: Original verses by Rudyard Kipling

FAR-OFF AMAZON
I’ve never sailed the Amazon,
I’ve never reached Brazil;
But the Don and Magdalena,
They can go there when they will!
Yes, weekly from Southampton
Great steamers, white and gold,
Go rolling down to Rio (Roll down – roll down to Rio!).
And I’d like to roll to Rio
Some day before I’m old!

I’ve never seen a Jaguar,
Nor yet an Armadill –
He’s dilloing in his armour,
And I s’pose I never will,
Unless I go to Rio
These wonders to behold –
Roll down -- roll down to Rio –
Roll really down to Rio!
Oh, I’d love to roll to Rio
Some day before I’m old!

HOMER
When ’Omer smote ’is bloomin’ lyre,
He’d ’eard men sing by land an’ sea;
An’ what he thought ’e might require,
’E went an’ took -- the same as me!

The market-girls an’ fishermen,
The shepherds an’ the sailors, too,
They ’eard old songs turn up again,
But kep’ it quiet -- same as you!

They knew ’e stole; ’e knew they knowed.
They didn’t tell, nor make a fuss,
But winked at ’Omer down the road,
An’ ’e winked back -- the same as us!

На далекой Амазонке: On far away Amazon

На далекой Амазонке
On far away Amazon
Не бывал я никогда.
I never visited.
Только "Дон" и "Магдалина" -
Only Don and Magdalene -
Быстроходные суда -
Fast-going ships
Только "Дон" и "Магдалина" -
Only Don and "Magdalene"
Ходят по морю туда.
Go on the sea there.
Из Ливерпульской гавани
From Liverpool harbor
Всегда по четвергам
Always on Thursdays
Суда уходят в плаванье
The ships leave to swim (on a voyage)
К далеким берегам.
To far away shores.
Плывут они в Бразилию,
They swim to Brazil,
Бразилию,
Brazil,
Бразилию.
Brazil.
И я хочу в Бразилию
And I want to go to Brazil
К далеким берегам!
To far away shores!

Никогда вы не найдете
You'll never find
В наших северных лесах
In our northern forests
Длиннохвостых ягуаров,
Long-tailed jaguars,
Броненосных черепах.
armored turtles.
Но в солнечной Бразилии,
But in sunny Brazil,
Бразилии моей,
Brazil mine,
Такое изобилие
(there is) Such abundance
Невиданных зверей!
[of] Never-seen beasts!
Увижу ли Бразилию,
Will I ever see Brazil,
Бразилию,

Brazil,
Бразилию,
Brazil,
Увижу ли Бразилию
Will I ever see Brazil
До старости моей?
Before my old age?

Гомер: Homer

Гомер все на свете легенды знал,
Homer all in the world legends knew,
И все подходящее из старья
And all suitable from old trash
Он, не церемонясь, перенимал,
He without hesitation adopted,
Но с блеском - и так же делаю я.
But with polish – and likewise do I.

А девки с базара да люд простой
And maids from the market as simple folk
И все знатоки из морской братвы
And all experts from the sea brotherhood
Смекали: новинки-то с бородой,
Understood: the new thing has a beard (the "new" story is old)
Но слушали тихо - так же, как вы.
But listened quietly - the same as you.

Гомер был уверен: не попрекнут
Homer was certain: [they] wouldn't accuse [ridicule]
За это при встрече возле корчмы,
For this, while meeting near a tavern,
А разве что дружески подмигнут,
But maybe with friendliness wink,
И он подмигнет - ну так же, как мы.
And he would wink – well, same as us [as we would].

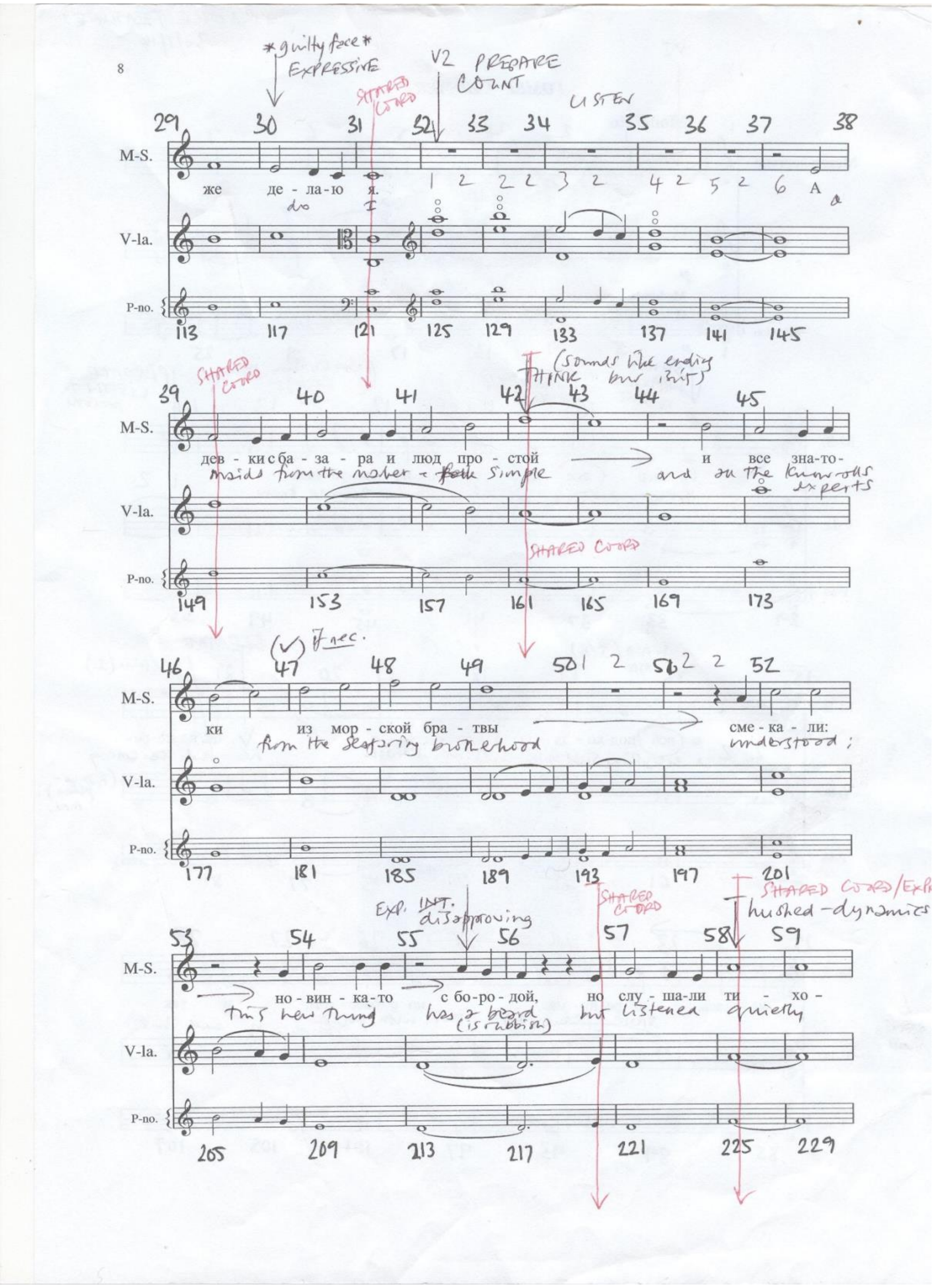

Figure B1. Page of singer's annotated score (rehearsal features for "Homer," p. 2).

Appendix C: Rehearsal features and performance cues (percentages represent numbers/rehearsal or performance).

Table C1. Rehearsal features and performance cues: "Amazon" (viola, memory).

		Rehearsal Count (%)	Perf. 1 Count (%)	Perf. 2 Count (%)	Perf. 3 Count (%)	Total Count (%)
Structural	Section	9 (11.8)			6 (6.3)	15 (5.1)
	Subsection		1 (1.59)			1 (0.34)
	Switch	2 (2.63)	2 (3.17)	2 (3.4)	5 (5.2)	11 (3.7)
All structural		11 (14.5)	3 (4.76)	2 (3.4)	11 (11.5)	27 (9.2)
Basic	Prepare	2 (2.63)		4 (6.8)	3 (3.1)	9 (3.1)
	Breath		5 (7.94)	5 (8.5)	3 (3.1)	13 (4.4)
	Word (B)	1 (1.32)				1 (0.34)
	Pitch	9 (11.8)	3 (4.76)	9 (15.3)	12 (13.0)	3 (1.0)
	Fingering				3 (3.1)	33 (11.2)
	Bowing				1 (1.0)	1 (0.34)
All basic		12 (15.8)	8 (12.7)	18 (30.5)	22 (22.9)	60 (20.4)
Interpretive	Word (I)	1 (1.32)	3 (4.76)		2 (2.1)	6 (2.0)
	Sound	5 (6.58)	1 (1.59)		5 (5.2)	11 (3.7)
	Tempo	10 (13.2)	2 (3.17)	4 (6.8)	10 (10.4)	26 (8.8)
	Dynamics	3 (3.95)	5 (7.94)	3 (5.0)	4 (4.2)	15 (5.1)
All interpretive		19 (25.0)	11 (17.5)	7 (11.9)	21 (21.9)	58 (19.7)
Expressive		6 (7.89)	4 (6.35)	7 (11.9)	10 (10.4)	27 (9.2)
Memory		6 (7.89)	15 (23.8)	2 (3.4)	11 (11.5)	34 (11.6)
Coordinate		12 (15.8)	5 (7.94)	6 (10.2)	14 (14.6)	37 (12.6)
Shared	Expressive	2 (2.63)	6 (9.52)	6 (10.2)	1 (1.0)	15 (5.1)
	Coordinate	8 (10.5)	11 (17.5)	11 (18.6)	6 (6.25)	36 (12.2)
All shared		10 (13.2)	17 (27.0)	17 (28.8)	7 (7.3)	51 (17.4)
	Total	76	63	59	96	294

Table C2. Rehearsal features and performance cues: "Amazon" (singer, score).

		Rehearsal Count (%)	Perf. 1 Count (%)	Perf. 2 Count (%)	Perf. 3 Count (%)	Total Count (%)
Structural	Section	2 (2.47)			2 (4.17)	4 (2.0)
	Subsection	21 (25.9)			10 (20.8)	31 (15.7)
	Switch					
All structural		23 (28.4)			12 (25.0)	35 (18.0)
Basic	Prepare	15 (18.5)	1 (2.94)	2 (5.71)	13 (27.1)	31 (15.7)
	Breath	9 (11.8)	6 (17.6)	6 (17.1)	3 (6.25)	24 (12.1)
	Word (B)	2 (2.47)	2 (5.88)	2 (5.71)		6 (3.0)
	Pitch	2 (2.47)				2 (1.0)
All basic		28 (34.6)	9 (26.5)	10 (28.6)	16 (33.3)	63 (32.3)
Interpretive	Word (I)	4 (4.94)	2 (5.88)	2 (5.71)	4 (8.3)	12 (6.1)
	Sound	2 (2.47)	4 (11.8)	4 (11.4)		10 (5.1)
	Tempo	5 (6.17)			3 (6.25)	8 (4.0)
	Dynamics	1 (1.23)	1 (2.94)	1 (2.86)		3 (1.5)
All interpretive		12 (14.8)	7 (20.6)	7 (20.0)	7 (14.6)	33 (16.9)
Expressive		6 (7.41)	1 (2.94)	1 (2.86)	5 (10.4)	13 (6.7)
Memory						
Coordinate		2 (2.47)			1 (2.08)	3 (1.5)
Shared	Expressive	2 (2.47)	6 (17.6)	6 (17.1)	1 (2.08)	15 (7.67)
	Coordinate	8 (9.88)	11 (32.4)	11 (31.4)	6 (12.5)	36 (18.2)
All shared		10 (12.3)	17 (50.0)	17 (48.6)	7 (14.6)	51 (26.2)
	Total	81	34	35	48	198

Table C3: Rehearsal features and performance cues: "Homer" (singer, memory).

		Rehearsal Count (%)	Perf. 1 Count (%)	Perf. 2 Count (%)	Perf. 3 Count (%)	Total Count (%)
Structural	Section	3 (4.6)				3 (1.6)
	Subsection					
	Switch				3 (8.1)	3 (1.6)
All structural		3 (4.6)			3 (8.1)	6 (3.3)
Basic	Prepare	17 (25.8)	13 (31.7)	12 (30.8)	12 (32.4)	54 (29.5)
	Breath	3 (4.6)	3 (7.3)	3 (7.7)	1 (2.7)	10 (5.5)
	Word (B)	4 (6.1)		2 (5.71)	2 (5.4)	6 (3.3)
	Pitch					
All basic		24 (36.4)	16 (39.0)	15 (38.5)	15 (40.5)	70 (38.3)
Interpretive	Word (I)	12 (18.2)	2 (4.9)	2 (5.13)	6 (16.2)	22 (12.0)
	Sound	1 (1.5)				1 (0.5)
	Tempo					
	Dynamics	1 (1.5)	1 (2.4)	1 (2.6)	1 (2.7)	4 (2.2)
All interpretive		14 (21.2)	3 (7.3)	3 (7.7)	7 (18.9)	27 (14.8)
Expressive		8 (12. 1)	7 (17.1)	7 (17.9)	1 (2.7)	23 (12.6)
Memory		7 (10.6)	1 (2.4)		5 (13.5)	13 (7.1)
Coordinate						
Shared	Expressive	1 (1.5)	1 (2.4)	1 (2.6)	1 (2.7)	4 (2.2)
	Coordinate	9 (13.6)	13 (31.7)	13 (33.3)	5 (13.5)	40 (21.9)
All shared		10 (15.2)	14 (34.1)	14 (35.9)	6 (16.2)	44 (24.0)
	Total	66	41	39	37	183

Table C4: Rehearsal features and performance cues: "Homer" (viola, score).

		Rehearsal Count (%)	Perf. 1 Count (%)	Perf. 2 Count (%)	Perf. 3 Count (%)	Total Count (%)
Structural	Section	4 (13.3)	3 (5.2)		5 (10.4)	12 (5.7)
	Subsection		3 (5.2)		1 (2.1)	4 (1.9)
	Switch					
All structural		4 (13.3)	6 (10.3)		6 (12.5)	16 (7.6)
Basic	Prepare		4 (6.9)		3 (6.3)	7 (3.3)
	Breath					
	Word (B)					
	Pitch		18 (31.0)	18 (24.0)	10 (20.8)	46 (21.8)
	Fingering	2 (6.7)	4 (6.9)	4 (5.3)	3 (6.25)	13 (6.2)
	Bowing		1 (1.7)	2 (2.7)		3 (1.4)
All basic		2 (6.7)	27 (46.6)	24 (32.0)	16 (33.3)	69 (32.7)
Interpretive	Word (I)					
	Sound	7 (23.3)	4 (6.9)	14 (18.7)	4 (8.3)	29 (13.7)
	Tempo	1 (3.3)	1 (1.7)		1 (2.1)	3 (1.4)
	Dynamics		1 (1.7)	3 (4.0)	1 (2.1)	5 (2.4)
All interpretive		8 (26.7)	6 (10.3)	17 (22.7)	6 (12.5)	37 (17.5)
Expressive		4 (13.3)	1 (1.7)	6 (8.0)	1 (2.1)	12 (5.7)
Memory						
Coordinate		2 (6.7)	4 (6.9)	14 (8.7)	13 (27.1)	33 (15.6)
Shared	Expressive	1 (3.3)	1 (1.7)	1 (1.3)	1 (2.1)	5 (1.9)
	Coordinate	9 (30.0)	13 (22.4)	13 (17.3)	5 (10.4)	40 (19.0)
All shared		10 (33.3)	14 (24.1)	14 (18.7)	6 (12.5)	44 (20.9)
	Total	30	58	75	48	211

Appendix D: Rehearsal features and performance cues (percentages represent numbers/all annotations per song).

Table D1. Rehearsal features and performance cues: "Amazon" (viola, memory).

		Rehearsal Count (%)	Perf. 1 Count (%)	Perf. 2 Count (%)	Perf. 3 Count (%)	Total Count (%)
Structural	Section	9 (3.1)			6 (2.0)	15 (5.1)
	Subsection		1 (0.34)			1 (0.34)
	Switch	2 (0.68)	2 (0.68)	2 (0.68)	5 (1.7)	11 (3.7)
All structural		11 (3.7)	3 (1.0)	2 (0.68)	11 (3.7)	27 (9.2)
Basic	Prepare	2 (0.68)		4 (1.4)	3 (1.0)	9 (3.1)
	Breath		5 (1.7)	5 (1.7)	3 (1.0)	13 (4.4)
	Word (B)	1 (0.34)				1 (0.34)
	Pitch	9 (3.1)	3 (1.0)	9 (3.1)	12 (4.1)	33 (11.2)
	Fingering				3 (1.0)	3 (1.0)
	Bowing				1 (0.34)	1 (0.34)
All basic		12 (4.1)	8 (2.7)	18 (6.1)	22 (7.5)	60 (20.4)
Interpretive	Word (I)	1 (0.34)	3 (1.0)		2 (0.68)	6 (2.0)
	Sound	5 (1.7)	1 (0.34)		5 (1.7)	11 (3.7)
	Tempo	10 (3.4)	2 (0.68)	4 (1.4)	10 (3.4)	27 (9.2)
	Dynamics	3 (1.02)	5 (1.7)	3 (1.0)	4 (1.4)	15 (5.1)
All interpretive		19 (6.5)	11 (3.7)	7 (2.4)	21 (7.1)	59 (19.7)
Expressive		6 (2.0)	4 (1.4)	7 (2.4)	10 (3.4)	27 (9.2)
Memory		6 (2.0)	15 (5.1)	2 (0.68)	11 (3.7)	34 (11.6)
Coordinate		12 (4.1)	5 (1.7)	6 (2.0)	14 (4.8)	37 (12.6)
Shared	Expressive	2 (0.68)	6 (2.0)	6 (2.0)	1 (0.34)	15 (5.1)
	Coordinate	8 (2.7)	11 (3.7)	11 (3.7)	6 (2.0)	36 (12.2)
All shared		10 (3.4)	17 (5.8)	17 (5.8)	7 (2.4)	51 (17.4)
Total		76	63	59	96	294

Table D2: Rehearsal features and performance cues; "Amazon" (singer, score).

		Rehearsal Count (%)	Perf. 1 Count (%)	Perf. 2 Count (%)	Perf. 3 Count (%)	Total Count (%)
Structural	Section	2 (1.0)			2 (1.0)	4 (2.0)
	Subsection	21 (10.6)			10 (5.1)	31 (15.7)
	Switch					
All structural		23 (11.6)			12 (6.1)	35 (17.7)
Basic	Prepare	15 (7.7)	1 (0.5)	2 (1.0)	13 (6.6)	31 (15.7)
	Breath	9 (4.6)	6 (3.0)	6 (3.0)	3 (1.5)	24 (12.1)
	Word (B)	2 (1.0)	2 (1.0)	2 (1.0)		6 (3.0)
	Pitch	2 (1.0)				2 (1.0)
All basic		28 (14.1)	9 (4.6)	10 (5.1)	16 (8.1)	63 (31.8)
Interpretive	Word (I)	4 (2.0)	2 (1.0)	2 (1.0)	4 (2.0)	12 (6.1)
	Sound	2 (1.0)	4 (2.0)	4 (2.0)		10 (5.1)
	Tempo	5 (2.5)			3 (1.5)	8 (4.0)
	Dynamics	1 (0.5)	1 (0.5)	1 (0.5)		3 (1.5)
All interpretive		12 (6.1)	7 (3.5)	7 (3.5)	7 (3.5)	33 (16.7)
Expressive		6 (3.0)	1 (0.5)	1 (0.5)	5 (2.5)	13 (6.6)
Memory						
Coordinate		2 (1.0)			1 (2.08)	3 (1.5)
Shared	Expressive	2 (1.0)	6 (3.0)	6 (3.0)	1 (2.08)	15 (7.7)
	Coordinate	8 (4.0)	11 (5.6)	11 (5.6)	6 (3.0)	36 (18.2)
All shared		10 (5.1)	17 (8.6)	17 (8.6)	7 (3.5)	51 (25.8)
	Total	81	34	35	48	198

Table D3: Rehearsal features and performance cues: "Homer" (singer, memory).

		Rehearsal Count (%)	Perf. 1 Count (%)	Perf. 2 Count (%)	Perf. 3 Count (%)	Total Count (%)
Structural	Section	3 (1.6)				3 (1.6)
	Subsection					
	Switch				3 (1.6)	3 (1.6)
All structural		3 (1.6)			3 (1.6)	6 (3.3)
Basic	Prepare	17 (9.29)	13 (7.1)	12 (6.6)	12 (6.6)	54 (29.5)
	Breath	3 (1.6)	3 (1.6)	3 (1.6)	1 (2.7)	10 (5.5)
	Word (B)	4 (2.2)		2 (1.1)	2 (1.1)	6 (3.3)
	Pitch					
All basic		24 (13.1)	16 (8.7)	15 (8.2)	15 (8.2)	70 (38.3)
Interpretive	Word (I)	12 (6.6)	2 (4.9)	2 (1.1)	6 (3.3)	22 (12.0)
	Sound	1 (0.5)				1 (0.5)
	Tempo					
	Dynamics	1 (0.5)	1 (0.5)	1 (0.5)	1 (0.5)	4 (2.2)
All interpretive		14 (7.7)	3 (1.6)	3 (1.6)	7 (3.4)	27 (14.8)
Expressive		8 (4.37)	7 (3.4)	7 (3.4)	1 (0.5)	23 (12.6)
Memory		7 (3.4)	1 (0.5)		5 (2.7)	13 (7.1)
Coordinate						
Shared	Expressive	1 (0.5)	1 (0.5)	1 (0.5)	1 (0.5)	4 (2.2)
	Coordinate	9 (4.9)	13 (7.1)	13 (7.1)	5 (2.7)	40 (21.9)
All shared		10 (5.5)	14 (7.7)	14 (7.7)	6 (3.3)	44 (24.0)
	Total	66	41	39	37	183

Table D4: Rehearsal features and performance cues: "Homer" (viola, score).

		Rehearsal Count (%)	Perf. 1 Count (%)	Perf. 2 Count (%)	Perf. 3 Count (%)	Total Count (%)
Structural	Section	4 (1.9)	3 (1.4)		5 (2.4)	12 (5.7)
	Subsection		3 (1.4)		1 (0.5)	4 (1.9)
	Switch					
All structural		4 (1.9)	6 (2.8)		6 (2.8)	16 (6.1)
Basic	Prepare		4 (1.9)		3 (1.4)	7 (3.3)
	Breath					
	Word (B)					
	Pitch		18 (8.5)	18 (8.5)	10 (4.7)	46 (21.8)
	Fingering	2 (0.95)	4 (1.9)	4 (1.9)	3 (1.4)	13 (6.2)
	Bowing		1 (1.7)	2 (0.95)		3 (1.4)
All basic		2 (0.95)	27 (12.8)	24 (11.4)	16 (7.6)	69 (26.2)
Interpretive	Word (I)					
	Sound	7 (3.3)	4 (1.9)	14 (6.6)	4 (1.9)	29 (13.7)
	Tempo	1 (0.5)	1 (0.5)		1 (0.5)	3 (1.4)
	Dynamics		1 (0.5)	3 (1.4)	1 (0.5%)	5 (2.4)
All interpretive		8 (3.8)	6 (2.8)	17 (8.1)	6 (2.)	37 (14.1)
Expressive		4 (1.9)	1 (0.5)	6 (2.8)	1 (0.5)	12 (4.56)
Memory						
Coordinate		2 (0.95)	4 (1.9)	14 (6.6)	13 (6.2)	33 (12.5)
Shared	Expressive	1 (0.5)	1 (0.5)	1 (0.5)	1 (0.5)	5 (1.9)
	Coordinate	9 (4.3)	13 (6.2)	13 (6.2)	5 (2.4)	40 (19.0)
All shared		10 (4.7)	14 (6.6)	14 (6.6)	6 (2.8)	44 (16.7)
	Total	30	58	75	48	211

Appendix E: Numbers of annotations.

Note: Column A represents locations of rehearsal features, B thoughts in Performance 1, C in Performance 2, and D in Performance 3. Rehearsal features also noted all three performances represent core performance cues; thoughts in B, C or D represent spontaneous thoughts unless noted in one or more subsequent performances, in which case they are functional performance cues.

Table E1. “Amazon” (viola, memory): numbers of annotations.

		Rehearsal features, core and non-core performance cues								Functional performance cues and spontaneous thoughts						
		A	AB	AC	AD	ABC	ABD	ACD	ABCD	B	BC	BD	BCD	C	CD	D
Structural	Section	3			6					0						
	Sub-section									1						
	Switch	1				1				1					1	4
Basic	Prepare				2									3	1	
	Breath										5					3
	Word (B)	1														
	Pitch	1			5			2	1	1	1			4	1	3
	Fingering															3
	Bowing															1
Interpretive	Word (I)	1								3						2
	Sound	2			3					1						2
	Tempo				7	1		1	1					1		1
	Dynamics	2						1		5				2		3
Expressive		2			3				1	3				6		6
Memory		1			4		1			11	1	2			1	3
Coordinate		5			6					1	3			3		8
Shared	Expressive	1				1				1	4			1		1
	Coordinate	5	1	1		2				3	4	1	1	2	1	3
Individual total		25	1	1	36	5	1	4	3	31	18	3	1	22	5	43
Total x occurrences		25	2	2	72	15	3	12	12	31	36	6	3	22	10	43

Table E2. “Homer” (singer, memory): numbers of annotations.

		Rehearsal features, core and non-core performance cues								Functional performance cues and spontaneous thoughts						
		A	AB	AC	AD	ABC	ABD	ACD	ABCD	B	BC	BD	BCD	C	CD	D
Structural	Section	3														
	Sub-section															
	Switch															3
Basic	Prepare	7			2	1	1		6		4		1			2
	Breath					2			1							
	Word (B)	4														2
	Pitch															
Interpretive	Word (I)	9			2	1					1					4
	Sound	1														
	Tempo															
	Dynamics								1							
Expressive		3				4			1	1	1			1		
Memory		6			1					1						4
Coordinate																
Shared	Expressive								1							
	Coordinate	3			1	1			4	8						
Individual total		36			6	9	1		14	2	14		1	1		15
Total x occurrences		36			12	27	3		56	2	28		3	1		15

Appendix F: Numbers and percentages of utterances (rehearsal talk), features, and thoughts in Performances 1, 2, and 3.

Table F1. "Amazon" (viola player, memory): Numbers and percentages of utterances (rehearsal talk), features, and thoughts in Performances 1, 2, and 3.

Categories	Utterances Count (%)	Features Count (%)	Performance 1 Count (%)	Performance 2 Count (%)	Performance 3 Count (%)
Structure	4 (7.1)	9 (11.8)	1 (1.6)	0	6 (7.5)
Switch	8 (14.3)	2 (2.6)	2 (3.2)	2 (3.3)	5 (6.3)
Pitch	5 (8.9)	9 (11.8)	3 (4.8)	9 (15.0)	0
Breath	2 (3.6)	0	5 (7.9)	5 (8.3)	3 (3.8)
Ensemble (RT) / (shared) co-ordinate (thoughts)	4 (7.1)	20 (26.3)	16 (25.4)	17 (28.3)	20 (25.0)
Words (basic)	2 (3.6)	1 (1.3)	0	0	0
Entries (RT) / prepare	1 (1.8)	2 (2.6)	0	4 (6.7)	3 (3.8)
Dynamics	3 (5.4)	3 (3.9)	3 (4.8)	3 (5.0)	4 (5.0)
Words (interpretive)	2 (3.6)	1 (1.3)	3 (4.8)	0	2 (2.5)
Tempo / rubato	5 (8.9)	10 (13.2)	2 (3.2)	5	10 (12.5)
Colour / sound	0	5 (6.6)	1 (1.6)	0	5 (6.3)
Expressive	3 (5.4)	8 (10.5)	10 (15.9)	13 (21.7)	11 (13.8)
Memory	17 (30.4)	6 (7.9)	15 (23.8)	2 (3.3)	11 (13.8)
	56	76	63	60	80

Table F2. "Amazon" (singer, reading from score): Numbers and percentages of utterances (rehearsal talk), features, and thoughts in Performances 1, 2, and 3.

Categories	Utterances Count (%)	Features Count (%)	Performance 1 Count (%)	Performance 2 Count (%)	Performance 3 Count (%)
Structure	11 (7.6)	23 (28.0)	0	0	12 (25.5)
Switch	7 (4.9)	0	0	0	0
Pitch	5 (3.5)	2 (2.4)	0	0	0
Breath	7 (4.9)	9 (11.0)	6 (17.6)	6 (17.1)	3 (6.8)
Ensemble (RT) / (shared) co-ordinate (thoughts)	7 (4.9)	10 (12.2)	11 (32.3)	11 (31.4)	6 (12.80)
Words (basic)	8 (5.6)	2 (2.4)	2 (5.9)	2 (5.7)	0
Entries (RT) / prepare	8 (5.6)	16 (19.5)	1 (2.9)	2 (5.7)	13 (27.7)
Dynamics	13 (9.0)	1 (1.2)	1 (2.9)	1 (2.9)	0
Words (interpretive)	39 (27.1)	4 (4.9)	2 (5.9)	2 (5.7)	4 (8.5)
Tempo / rubato	12 (8.3)	5 (6.1)	0	0	3 (6.8)
Colour / sound	4 (2.8)	2 (2.4)	4 (11.8)	4 (11.4)	0
Expressive	9 (6.3)	8 (9.8)	7 (20.6)	7 (20.0)	6 (12.8)
Memory	14 (9.7)	0	0	0	0
	144	82	34	35	47

Table F3. “Amazon” (singer, memory): Numbers and percentages of utterances (rehearsal talk), features, and thoughts in Performances 1, 2, and 3.

Categories	Utterances Count (%)	Features Count (%)	Performance 1 Count (%)	Performance 2 Count (%)	Performance 3 Count (%)
Structure	0	3 (4.5)	0	0	0
Switch	2 (1.8)	0	0	0	3 (8.1)
Pitch	4 (3.6)	0	0	0	0
Breath	3 (2.7)	3 (4.5)	3 (7.3)	3 (7.7)	1 (2.7)
Ensemble (RT) / (shared) co-ordinate (thoughts)	9 (8.2)	9 (13.6)	13 (31.7)	13 (33.3)	5 (13.5)
Words (basic)	2 (1.8)	4 (6.1)	0	0	2 (5.4)
Entries (RT) / prepare	14 (12.7)	17 (25.8)	13 (31.7)	12 (30.8)	12 (32.4)
Dynamics	13 (11.8)	1 (1.5)	1 (2.4)	1 (2.6)	1 (2.7)
Words (interpretive)	24 (21.8)	12 (18.2)	2 (4.9)	2 (5.1)	6 (16.2)
Tempo / rubato	8 (7.3)	0	0	0	0
Colour / sound	3 (2.7)	1 (1.5)	0	0	0
Expressive	3 (2.7)	9 (13.6)	8 (19.5)	8 (20.5)	2 (5.4)
Memory	25 (22.7)	7 (10.6)	1 (2.4)	0	5 (13.5)
	110	66	41	39	37

Table F4. “Homer” (viola player, reading from the score): Numbers and percentages of utterances (rehearsal talk), features, and thoughts in Performances 1, 2, and 3.

Categories	Utterances Count (%)	Features Count (%)	Performance 1 Count (%)	Performance 2 Count (%)	Performance 3 Count (%)
Structure	3 (9.1)	4 (11.4)	6 (14.3)	0	7 (15.2)
Switch	2 (6.1)	0	0	0	0
Pitch	2 (6.1)	9 (25.7)	18 (42.9)	18 (29.5)	10 (21.7)
Breath	0	0	0	0	0
Ensemble (RT) / (shared) co-ordinate (thoughts)	9 (27.3)	9 (25.7)	7 (16.7)	18 (29.5)	18 (39.1)
Words (basic)	0	0	0	0	0
Entries (RT) / prepare	5 (15.2)	0	4 (9.5)	0	3 (6.5)
Dynamics	1 (3.0)	0	1 (2.4)	3 (4.9)	1 (2.1)
Words (interpretive)	1 (3.0)	0	2 (4.9)	0	0
Tempo / rubato	4 (12.1)	1 (2.9)	0	0	1 (2.1)
Colour / sound	0	7 (20.0)	4 (9.5)	14 (23.0)	4 (8.7)
Expressive	1 (3.0)	5 (14.3)	1 (2.4)	8 (13.11)	2 (4.4)
Memory	5 (15.2)	0	0	0	0
	33	35	42	61	46
